# Post Processing Strategies for the Enhancement of Mechanical Properties of ENMs (Electrospun Nanofibrous Membranes): A Review

**DOI:** 10.3390/membranes11010039

**Published:** 2021-01-05

**Authors:** Saad Nauman, Gilles Lubineau, Hamad F. Alharbi

**Affiliations:** 1 COHMAS Laboratory, Physical Sciences and Engineering Division (PSE), King Abdullah University of Science and Technology (KAUST), Thuwal 23955-6900, Saudi Arabia; 2 MS&E Department, Institute of Space Technology, Islamabad 44000, Pakistan; 3 Mechanical Engineering Department, King Saud University, P.O. Box 800, Riyadh 11421, Saudi Arabia; harbihf@ksu.edu.sa

**Keywords:** post-processing strategies, electrospinning, electrospun nanofibrous membrane, mechanical properties

## Abstract

Electrospinning is a versatile technique which results in the formation of a fine web of fibers. The mechanical properties of electrospun fibers depend on the choice of solution constituents, processing parameters, environmental conditions, and collector design. Once electrospun, the fibrous web has little mechanical integrity and needs post fabrication treatments for enhancing its mechanical properties. The treatment strategies include both the chemical and physical techniques. The effect of these post fabrication treatments on the properties of electrospun membranes can be assessed through either conducting tests on extracted single fiber specimens or macro scale testing on membrane specimens. The latter scenario is more common in the literature due to its simplicity and low cost. In this review, a detailed literature survey of post fabrication strength enhancement strategies adopted for electrospun membranes has been presented. For optimum effect, enhancement strategies have to be implemented without significant loss to fiber morphology even though fiber diameters, porosity, and pore tortuosity are usually affected. A discussion of these treatments on fiber crystallinity, diameters, and mechanical properties has also been produced. The choice of a particular post fabrication strength enhancement strategy is dictated by the application area intended for the membrane system and permissible changes to the initial fibrous morphology.

## 1. Introduction

Fabrication of nanoscale materials has attracted a lot of attention in recent years due to the promise of nanotechnology in various domains, owing to their unique mechanical, electrical, and optical properties distinct from their bulk counterparts [1,2]. A lot of effort has been invested in the control of size and morphology at the nanoscale in order to design and fabricate viable structures and devices [3] using so called bottom up and top down methods [4,5,6,7].

**Figure 1:**
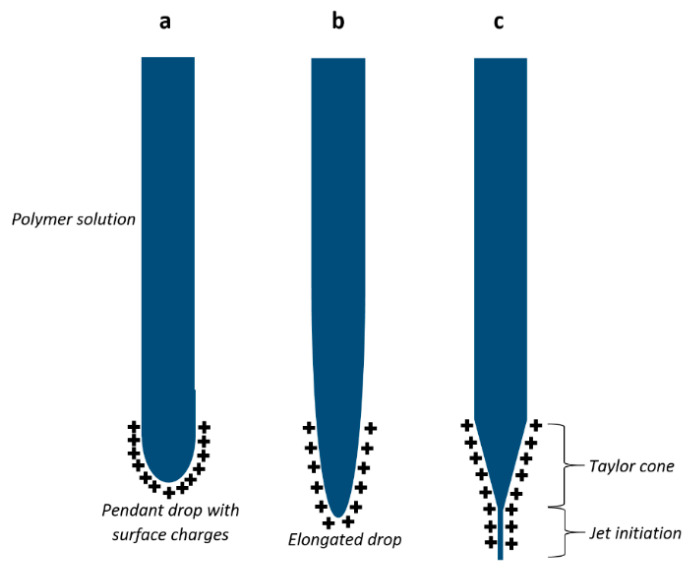
Different steps involved in Taylor cone formation: (**a**) Charges from the needle travel to the pendant polymer droplet, (**b**) the pendant drop gets elongated under the influence of electrostatic force, (**c**) the pendant drop further elongates into the form of a Taylor cone to initiate the jet (inspired and redrawn from [11]).

Electrospinning is one such promising technique for the fabrication of nanoscale fibers and their structures due to its simple execution. The versatility of this technique makes it suitable for many different polymeric materials, their blends, and composites in different forms such as solutions, sols, and polymer melts. This technique allows the drawing of a polymer solution or melt under the influence of an electrostatic force. This essentially results in the formation of polymer fibers when the stretching action is accompanied by solvent evaporation or polymer solidification. A basic electrospinning set-up is comprised of a polymer container with an attached needle and a conductive collector placed such that it faces the needle. Both the needle and the conductive collector are connected to the two terminals of a high voltage power supply. At high applied voltages, the droplet at the needle tip gets charged as charges travel from the conductive needle tip towards the polymer solution/melt. The electrostatic attraction of the collector causes the polymer solution/melt droplet to stretch and to assume a conical configuration, known as a Taylor cone [8]. Schematic representation of different steps involved in the development of a Taylor cone are given in Figure 1. The electrostatic forces should be large enough to overcome the surface tension of the polymer solution/melt in the Taylor cone. At the same time, the cohesive forces in the polymer solution/melt should be sufficient to allow it to be drawn towards the collector in the form of a continuous jet of polymer. During this drawing step, the solvent evaporation/polymer solidification is essential, as it results in the formation of a fiber. The charges transfer to the fiber surface and current flow mechanism changes from ohmic to convective. An important feature of polymer fiber formation in the convective region is the bending instability caused by repulsion between similarly charged fiber bends or loops. This bending instability, or whipping action, as it is called, causes further stretching and thinning of the polymer fiber before it is finally deposited on the surface of the collector as an interconnected web of fibers [9,10].

**Figure 2:**
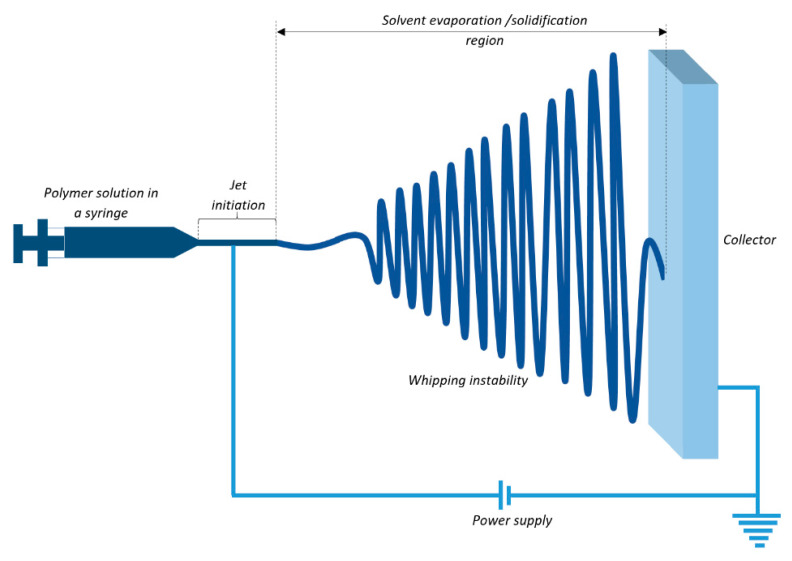
A schematic of a typical electrospinning setup with its various components.

A functional electrospinning set-up is typically comprised of a syringe pump with a metallic needle, a metallic collector, and a D.C. power supply in the range from 5–50 kV. The syringe pump continuously pumps the polymer solution or melt to the needle for uninterrupted fiber collection on the collector, which is usually grounded. Different components of a typical electrospinning set-up are schematically shown in Figure 2.

Over the years, the basic electrospinning mechanism has evolved into more complex and polyvalent systems capable of handling more than one type of material in solution or molten forms. This has been made possible by bringing innovations in either the needle design or ejector configuration. These systems include coaxial electrospinning, where a core shell type fiber can be electrospun with different materials in the core and shell. There is a possibility of electrospinning hollow fibers as well using this technique. Similarly, multiaxial electrospinning allows the handling of more than two materials with fiber being comprised of many sheaths or laminae around a central core. An example of innovative ejector configuration can be cited in the form of needleless electrospinning. It allows electrospinning directly from an open liquid surface. A great advantage of this technique is its high production rate suitable for commercial applications.

Many applications of electrospun fibers and membranes include biomedical (tissue engineering [12,13,14], drug delivery [15,16,17], immobilization of enzymes [18,19,20], wound dressing [21,22,23] and antibacterial membranes [24,25,26]), textiles [27,28,29], separation membranes (Li ion battery separators [30,31,32], distillation [33,34,35] and filtration membranes [36,37,38]), sensors [39,40,41], and high performance composite materials (reinforcing agents [42,43,44] or vascular networks of healing agents [45,46,47]), etc.

## 2. Electrospinning Parameters and Their Influence on Mechanical Properties of Nanofibrous Membranes

In order to optimize mechanical properties of electrospun fibers and their membranes, the following objectives need to be achieved during the execution of electrospinning process:

1. Uniform diameter and morphological homogeneity of fibers;
2. defect (beads and ribbons) free fiber collection;
3. stretching of fibers, thus improving their crystallinity and mechanical properties while reducing their diameters and associated defects; and
4. continuous and preferably aligned fiber deposition on the collector.

In order to help meet the above stated conditions, the following aspects of an electrospinning process need precise control:

1. Processing parameters;
2. solution/melt characteristics;
3. environmental factors; and
4. collector configuration.

The electrospinning is only successful for a certain range of carefully calibrated processing parameters, solution characteristics, and environmental factors. This range can be termed as the processing window or optimum range beyond which electrospinning is not possible.

**Processing parameters** include applied electric field, distance between the needle tip and the collector, solution feed rate and needle diameter, etc. As the stretching force is directly proportional to the applied electric field during the flight of the electrospinning jet, the fiber diameter generally reduces with the increase in applied potential difference between the needle and collector [48]. This is true for the processing window only, as when the applied voltage is increased beyond this range, bead formation has been observed. This is due to the increase in jet velocity without a proportional increase in feed rate causing a disruption in the jet formation and shorter time available for the solvent to evaporate [49]. Similarly, when the solution feed rate is increased beyond its optimum processing window, defective fibers with beads and ribbon like morphology are formed due to incomplete solvent evaporation from the jet surface. An increase in feed rate also increases the electric current, resulting in the reduction of surface charge density leading to fiber merger on their way towards the collector due to decreased repulsive forces between the individual fibers. This is termed as garland formation. As the distance between the needle tip and the collector is increased, greater stretching forces are experienced by the advancing fibers, especially in the whipping area due to bending instabilities leading to a reduction in fiber diameters [50].

**Solution characteristics** in the case of solution electrospinning include choice of the particular solvent, polymer concentration in the solvent, and solution conductivity. The choice of a particular solvent for electrospinning is based firstly on its compatibility with the polymer, i.e., the polymer should be soluble in the solvent and secondly on the basis of its boiling point, which determines its volatility. A moderate boiling point of a solvent would ensure its rapid evaporation before deposition of the fibers on the collector while avoiding clogging of the needle due its sufficiently low volatility [51]. This would ensure smooth operation of electrospinning process for the deposition of defect free continuous fibers. The concentration of polymer in the solvent will directly impact the viscosity of the solution. A high concentration viscous solution results in the formation of large diameter bead-less fibers due to greater entanglement of polymer chains per unit fiber cross section [52]. When the polymer concentration is reduced below a certain optimum value, the polymer jet fragments on its way towards the collector due to low viscosity and lack of cohesion, resulting in the formation of beaded and discontinuous fibers. Solution conductivity determines surface charge density. Surface charges allow electrostatic interaction between the droplet at the needle tip and the collector. This results in the formation of Taylor cone, which is the beginning of electrospinning process. An increase in solution conductivity also increases the repulsive interaction between the bends of fibers, intensifying the whipping action, causing stretching and eventual thinning of fibers [53].

For melt electrospinning, the polymer melt instead of polymer solution is directed towards the collector under the influence of electrostatic force. The parameters which control the deposition of fibers in melt electrospinning include temperature, which ensures melting of the polymer and jet formation under the stretching force, and flow rate, which depends on the temperature maintained during electrospinning. Higher flow rates result in larger fiber diameters. Since no solvent evaporation is involved, unlike solution electrospinning, yields could be significantly higher even at low flow rates. Molecular weights are an important concern for melt electrospinning. Low molecular weights result in discontinuous electrospinning adversely affecting the fiber properties. On the other hand, excessively high molecular weights also hamper continuous flow and deposition of polymer on the collector. A blend of low molecular weight and high molecular weight polymers may be used in order to achieve requisite flow rates. Cooling rate is another factor for optimum fiber deposition, as melt electrospun fibers rely on cooling for solidification as against evaporation in the case of solution electrospinning.

**Environmental factors** include relative humidity and ambient temperature. The effect of relative humidity on fiber morphology depends on polymer hydrophobicity, solvent miscibility with water, and solvent volatility [54]. Fiber diameter might increase or decrease with relative humidity depending on the nature of the polymer [55]. An increase in ambient temperature has a two-pronged effect on electrospinning; on one hand, it reduces the viscosity of the solution, while on the other hand, it helps increase the evaporation rate of the solvent, which results in rapid solidification of the fiber jet. The synergistic effect of these two mechanisms on the advancing jet is to reduce the fiber diameters [55].

**Collector configuration** is an important factor which not only determines whether the fibers are deposited in random or aligned fashion, but can also help draw fibers with associated reduction in diameters, enhancement in crystallinity, and mechanical properties. As such, collection of fibers is the last step in the electrospinning process which can affect mechanical properties of the produced fibers. In its most rudimentary form, a collector is a conductive plate which is usually electrically ground and is used for the collection of a web of random fibers. Since these fibers are deposited over one another in random fashion, such a collector does not induce any stretching action on the collected fibers. An improved collector configuration is that of a rotating drum, which is essentially made of a conductive material or at least coated with one and is usually connected to electrical ground. This kind of collector can not only stretch the deposited fibers, but also ensures a certain degree of alignment of collected fibers in the direction of drum rotation. The draw ratios applied, and to a certain extent alignment of fibers depend on the surface speed of the rotating drum with higher speeds, inducing greater stretch and orientation until fiber failure initiates due to excessive tensile force. Various modifications to the original plate collector and drum collector designs exist which modify the electric field near the collector and help improve the alignment of fibers. These include parallel electrodes [56], a rotating wire drum collector [57], a rotating tube collector with knife-edge electrodes below it, an array of counter electrodes [58], a disc collector [59], a counter-electrode [60], a rotating tube collector with knife-edge electrodes below [61], a rotating drum with a sharp pin inside [62], parallel ring collectors [63], and a conveyor belt made of an insulation material sliding on the top of the grounded collector plate underneath [64], etc.

## 3. Mechanical Characterization of Nanofibrous Membranes

As discussed above, the electrospinning process and material parameters together with the collector configuration and ambient conditions have an important bearing on the mechanical properties of electrospun fibers and their membranes. Once the electrospinning process is complete, the mechanical properties of the collected fibers can be studied to assess the impact of aforementioned parameters by removing either individual fibers or the membrane specimens from the collector. Two mechanical testing protocols can therefore be identified in the literature:

1. Single fiber mechanical tests, or
2. membrane mechanical tests.

**Single fiber mechanical tests** can be difficult to execute, partly because of the difficulty in isolating and handling these fine fibers [65] and partly because of the high resolutions needed for the actuators and load cells for testing these ultrafine fibers [66]. These tests can be classified as tensile, bending, and indentation tests.

Tensile tests for submicron scale ultrafine fibers are challenging because of the difficulty in properly gripping a single fiber and its alignment on the fixture along the loading direction. At the same time these tests can give valuable information about the mechanical properties of single fibers and can be used as true indicators of the effect of various processing parameters during the electrospinning process.

For tensile testing, AFM (Atomic Force Microscopy) cantilevers are usually attached to the fiber, which is stretched by means of the stepper motor of AFM system, while the test is conducted in scanning electron microscope (SEM) chamber [67]. In certain cases where the fiber size warrants, an optical microscope may be used instead of electron microscope [68]. In this case, the tensile load is applied by the displacement of microscope stage. The force is measured by the deflection of the soft cantilever. AFM systems are particularly suitable for mechanical testing of single nanofibers because of their high resolution and accuracy. A slight variation of this approach uses a substrate such as a silicon wafer to which one end of the fiber is attached using a glue [69]. The AFM tip acts as the deflection element to which the other end of the fiber is connected. As the spring constant of the cantilever is known, its deflection gives the measure of tensile forces applied on the fiber. The strain in the fiber is determined through simultaneous SEM or optical microscopy.

An important variation in the traditional single fiber tensile test is the development of three point tensile test, where the cantilever apex tip stretches the fiber attached to TEM (Transmission Electron Microscopy) grids with an adhesive by dragging it laterally [70].

An alternative approach of tensile testing fibers could be through the use of a commercially available single fiber tensile testing system such as Nano Bionix^®^ and Nano UTM^®^. The specimen could be prepared by directly depositing the fibers on a cardboard [71] or metal frame [72], mounting it on the nanotensile tester, and cutting the cardboard frame across the two vertical sides to allow tensile loading of fibers. In certain cases, fibers can be directly electrospun over the TEM grids to avoid complexities associated with fiber placement and manipulation. Customized equipment for fiber testing has also been developed by various researchers [71].

Bending tests can also be performed on single fibers by positioning single fibers over a grooved substrate [73]. Specific instances of such tests include; a nanofiber suspended over an etched groove in silicon wafer with an AFM tip applying deflection at the center point to conduct three-point bending test [74], a nanofiber fixed at one end with epoxy while the other end is deflected using the AFM tip to conduct two point bending test [75], application of force at different points along the length of a suspended fiber to measure fiber deflection to conduct a multi-point bending test [73].

Nanoindentation is another technique which can be employed to study elastic and elastic–plastic behavior of nanofibers using a specially built AFM based nanoindentation system or an AFM tip [76]. The image of the indentation area immediately after the indentation helps calculate the projected area for determination of hardness and elastic modulus.

**Membrane mechanical tests** using the uniaxial tensile testing equipment gives homogenized macromechanical properties of the web of electrospun fibers. These properties are dependent on the fiber arrangement, membrane morphology, fiber packing density, and inter-fiber interaction in addition to the structural properties of the individual fibers. It is common practice to conduct uniaxial tensile tests on rectangular membrane specimens of electrospun fibers following ASTM D882–18 [77]. In addition to uniaxial tensile testing, Dynamic Mechanical Analysis (DMA) is also sometimes employed to determine viscoelastic properties of electrospun membranes [78,79].

For membrane testing, the rectangular specimens have to be cut from the web of fibers removed from the collector. Given the fragility of the deposited electrospun membranes, their removal from the collector could be difficult. To facilitate removal of the electrospun membrane from the collector, a non-stick Teflon coating may be applied on the conductive collector, alternatively a separation medium such as gelatin, alginate, or oil may be placed/deposited on the collector surface [80]. The water-soluble separation media enables easy peel off when soaked in water.

A difference of three orders of magnitude in Young’s moduli of the membranes tested at the macro-scale compared with the single fibers tested using the AFM assisted testing was reported for polycaprolactone (PCL) scaffolds (3.8 ± 0.8 MPa for the membrane against 3.7 ± 0.7 GPa for individual PCL fibers) [73]. The ‘lacunar’ structure of high porosity random fiber mats was dubbed as the reason for this disparity in the mechanical properties.

Given the simplicity of sample preparation and procedure, macro scale tensile testing of membrane specimens is common across the literature. The reasons for its popularity include the rapid and meaningful insights that this test provides into the effect of post processing techniques on the mechanical properties of electrospun membranes, as will be explained in the following section.

**Figure 3:**
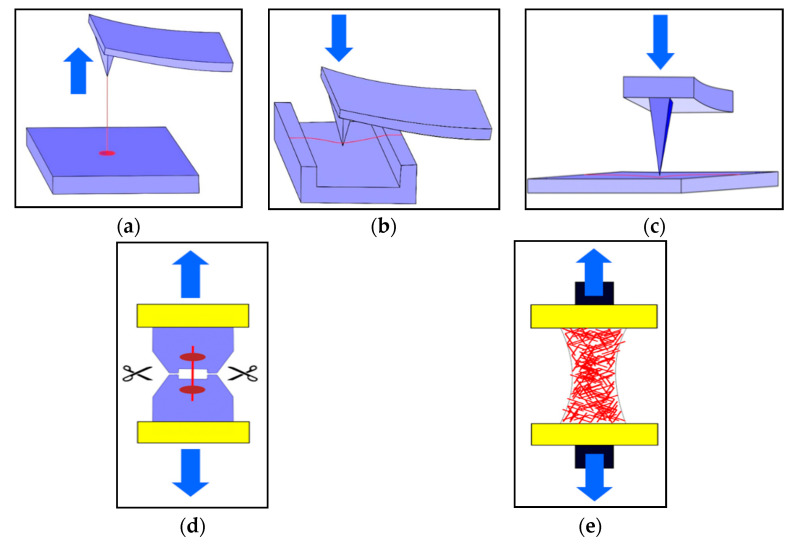
Schematic description of single fiber and macro scale mechanical testing protocols employed for electrospun nanofibrous membranes (ENMs), (**a**) AFM cantilever assisted tensile test, (**b**) AFM cantilever assisted bending test, (**c**) AFM cantilever assisted indentation test, (**d**) single fiber tensile test, (**e**) and membrane tensile test.

A schematic description of both the single fiber and macro scale mechanical testing protocols employed for electrospun nanofibrous membranes (ENMs) is given in Figure 3.

## 4. Post Fabrication Techniques for the Enhancement of Mechanical Properties of ENMs

**Figure 4:**
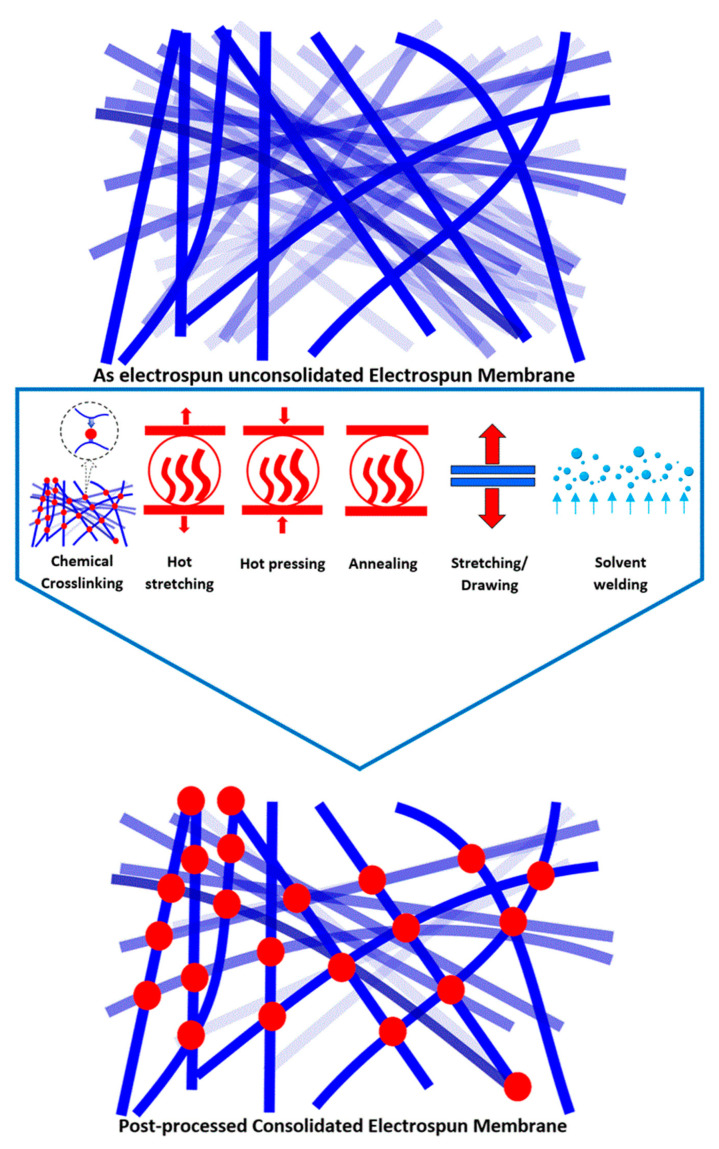
Schematic illustration of various post fabrication techniques employed for the enhancement of mechanical properties of ENMs (Unconsolidated membranes are those which have not yet undergone post-processing treatment, while consolidated ones are post-processed membranes).

As remarked by Kaur et al. [81], the electrospun membranes are in a ‘cotton like’ state when spun with little inter-fiber cohesion and structural integrity. These are difficult to handle and unsuitable for many practical applications. Electrospun membranes therefore need to be post processed in order to transform them from a ‘loose cotton like to paper like’ morphology with enhanced structural integrity permitting their practical exploitation in various domains. A schematic illustration of various post fabrication techniques found in the literature has been given in Figure 4.

In the following section, a survey of the techniques used for the enhancement of mechanical properties of electrospun membranes has been produced.

### 4.1. Crosslinking of ENMs

Crosslinking of polymers has been widely used to produce thermosetting polymers. It has been widely reported that the crosslinking of polymers results in the formation of a 3D macromolecular network. An important consequence of network formation is the gelation of polymer, which increases its viscosity. This is accompanied by an increase in the stiffness as polymeric chains become integrally bound to one another while elongation at break is generally compromised for the same reason. Crosslinking can be classified into physical and chemical crosslinking. Physical crosslinking depends on many factors including hydrogen bonding, electrostatic interaction between ions, as well as crystallization acting as binding points between molecules. Chemical crosslinking results when covalent bonds are formed between the molecular chains of a polymer, thus increasing its molecular weight and improving mechanical properties such as strength, stiffness, abrasion resistance, hardness and thermal stability, etc. The kinetics of a crosslinking reaction are dictated by the chemical structure of the reactants involved and their concentration [82]. Therefore, crosslink density and rate of reaction can be controlled by varying the concentration of crosslinker and reaction conditions. Similar crosslinking strategies can be adopted for electrospun membranes using a suitable crosslinker in order to create inter-fiber bonds. Crosslinking in the membrane structures is expected to bond the fibers at their crossover points, restricting their movement and slippage. This paves the way for their application in various domains where structural integrity and stable mechanical properties cannot be compromised.

Li et al. [83] used the natural crosslinker Genipin (GP), because of its low cytotoxicity, for crosslinking chitosan (CTS) nanofibrous membranes intended for use as scaffolds in tissue engineering applications. They blended CTS with fiber forming additive polyethylene oxide (PEO) in different mass ratios prior to electrospinning. The electrospun nanofibrous membrane obtained from a CTS/PEO blend with the mass ratio of 85/15 was placed in PBS (phosphate buffer solution) containing different concentrations of GP (0.1%, 0.5%, and 1% *w*/*v*) at 37 °C for crosslinking. The time of exposure was varied from 6 h, 12 h, to 24 h. At the end of the designated exposure duration, the membranes were washed in PBS and immersed in deionized water for 2 h and then in ethanol for 12 h in order to terminate the crosslinking process. In order to gauge the impact of crosslinking on the performance of nanofibrous membranes, tensile tests in both the dry and wet states were carried out on the specimens having dimensions of 5 cm × 1 cm at a constant crosshead speed of 30 mm/min. It was reported that crosslinking improved the stiffness in the dry state, but the ultimate tensile strength of the crosslinked membrane was considerably lower than that of the pristine nanofibrous membrane (5 MPa against 14 MPa) owing to the brittleness and reduced elongation at break after crosslinking. The wet state tensile properties were considerably better with 0.5% GP crosslinked CTS membrane, exhibiting the greatest strength retention ratio of (dry to wet) of 84.19%, indicating an optimized crosslinker concentration for the CTS membranes.

Wang and coworkers [84] have reported a crosslinking and surface coating mechanism for PVA scaffolds intended for high flux ultrafiltration membranes since the uncrosslinked PVA is water soluble and is unsuitable for use in water filtration applications. Three categories of PVA membranes were prepared, i.e., high, medium, and low molecular weight variants. In order to induce crosslinking, they immersed the PVA membranes in glutaraldehyde (GA) solution for 24 h, and were then washed in water repeatedly before being dried in a hood. The mechanical properties were determined by cutting rectangular specimens of 20 mm × 5 mm × 10 mm (length × width × thickness). Gauge length of the specimens was 10 mm, and crosshead speed was 2 mm/min during displacement controlled tensile tests. The mechanical properties of the 95% hydrolyzed high molecular weight (85,000–124,000 g/mol) PVA membranes were reported to improve significantly with tensile modulus and strength, increasing from 40 MPa to 48 MPa and from 7.6 MPa to 13.5 MPa, respectively. As expected, crosslinking reduced the elongation at break from 130% to 67%. The fiber diameter remained almost the same before and after crosslinking (~230 nm), though crosslinking-induced volume shrinkage (<5%) was reported.

A filtration membrane system comprising of a mid-layer of polyacrylonitrile (PAN) electrospun nanofibrous membrane coated with crosslinked PVA top layer to design high flux thin film nanofibrous composites (TFNCs) was reported by Yoon and coworkers [85]. Crosslinked polyvinyl alcohol (PVA) served as a thin hydrophilic barrier layer with low hydraulic resistance and fouling potential. In order to create the barrier layer of PVA, first its aqueous solution was prepared. After adjusting its pH by the addition of 1.2 M HCl, the glutaraldehyde (GA) solution with the concentration ratio [-OH/GA] (hydroxyl groups in PVA to GA) of 4 was added. The PVA/GA solution was then cast over the PAN membrane. Aldehyde groups in GA react with the hydroxyl groups of PVA to create a crosslinked structure [82]. The reaction is catalyzed by the acid. The reaction completed by keeping the composite film in a humid chamber for 12 h and then washing it with water. In order to gauge the impact of crosslinking on the properties of the TFNC, the authors subjected it to a cross-flow filtration test using a model oil in water emulsion. It was reported that the TFNC with crosslinked PVA barrier coating demonstrated good mechanical integrity, as it sustained typical ultrafiltration pressure ranging from 50–150 psig. The flux properties of the developed TFNCs were compared with two commercially available PAN-UF (ultrafiltration) membranes (PAN10 and PAN400). In terms of the filtration performance of oily water, the developed TFNCs demonstrated 12 times higher permeate flux than PAN10 after 190 h of continuous cross-flow operation.

**Figure 5:**
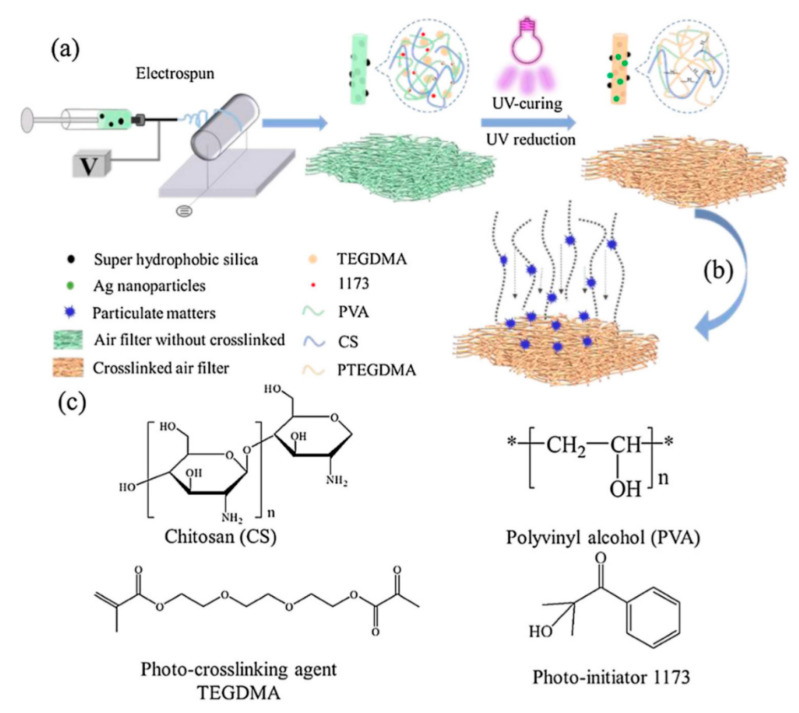
Fabrication process for antibacterial and hierarchical CS/PVA (Chitosan/polyvinyl alcohol) nanofibrous membranes by combination of (**a**) electrospinning, one step UV reduction and cured, (**b**) filtration process of the CS-PVA@SiO_2_ NPs-Ag NPs air filtration membranes, and (**c**) the chemical structure of CS/PVA/TEGDMA/1173 [24] (reprinted with permission from Elsevier).

Another application of CS/PVA (Chitosan/polyvinyl alcohol) crosslinked structures was presented by Zhu et al. [24] when they added hydrophobic SiO_2_ nanoparticles in the CS-PVA solution as well as the silver nanoparticles to endow these membranes with antibacterial properties. These membranes were intended for air filtration applications in personal protective masks. The composite electrospun membranes were exposed to UV light for six hours to obtain semi-interpenetrating polymer networks (SIPNs). A complete route of the electrospinning and crosslinking process is schematically shown in Figure 5. Even though the effect of crosslinking was not evaluated using mechanical testing procedures, the higher filtration efficiencies and integrity are expected in part due to hierarchical structure and partly due to integrity owing to crosslinking.

Hybrid structures made of different types of electrospun membranes in different layers can be treated by adopting different post fabrication treatment methodologies for each of the separate layers. Recently Tian and coworkers [86] have reported a superwetting composite membrane for oil in water emulsion separation processes in order to mitigate membrane fouling and clogging. Coarse PAN fibers were first electrospun on non-woven PET (Polyethylene terephthalate) substrate using 8 wt.% PAN solution in DMF (Dimethylformamide). A thinner layer of finer fibers was then deposited on the coarse fiber layer using 5 wt.% PAN solution using the same electrospinning equipment. After the deposition of double layer on the substrate, the resulting laminate was then hot pressed at 100 °C to improve mechanical stability of the nanofibrous membrane while preserving its fibrous morphology. The hot pressing temperature (100 °C) was selected to be slightly greater than the glass transition temperature (96 °C) and much lower than the fusion temperature (322 °C) of the material. The electrospun membrane was saturated with water in order to fill the pores. Excessive water was subsequently removed by using a rubber roller. The wet PAN ENM was then sprayed with solution of CNTs/PVA (0.2 wt.% CNTs and 0.05 wt.% PVA) and 0.05 wt.% Glutaraldehyde (GA) using a spray coating machine. The membrane was then crosslinked by curing at 60 °C for 20 min in order to stabilize the CNTs/PVA on the surface. It was reported that the combined impact of superior mechanical properties of surface deposited CNTs and crosslinking of PVA was an increase in tensile modulus of the membrane by 20%.

Satilmis and Uyar [87] reported crosslinking of Hydrolyzed Polymers of Intrinsic Microporosity (HPIM), PIM-1 in this case with polybenzoxazine (BA-a) for water treatment applications in filtration media. Fifty percent *w*/*v* solutions of HPIM were prepared and blended with BA-a monomer (10, 25, 33% *w*/*w*) in DMF at room temperature. The solutions were used for electrospinning of nanofibrous membranes. Thermally assisted crosslinking of the HPIM with BA-a was carried out by stepwise heating of membranes at 150 °C, 175 °C, 200 °C, and 225 °C successively for one hour each. The heating profile ensured the ring opening of BA-a and its subsequent crosslinking with HPIM in the nanofibrous membrane. Dynamic mechanical analysis (DMA) was used to quantify the effect of crosslinking on the stability of membranes. Specimens having dimensions of 8 mm × 6 mm × 0.1 mm were tested in force-controlled mode. Force was ramped at a rate of 0.05 N/min. Young’s modulus of HPIM, as calculated from the initial region of stress–strain plots, increased from 16 MPa to 67 MPa due to crosslinking. Direct correlation was found between the crosslinker content and Young’s modulus, tensile strength, and storage modulus as their values progressively increased when the BA-a content was increased from 10%, 25%, to 33% *w*/*w*.

Another water-soluble natural polymer which can be stabilized by crosslinking is gelatin. Jalaja et al. [88] reported scaffolds intended for tissue engineering applications made of electrospun nanofibrous gelatin. They crosslinked the gelatin nanofibrous mats with dextran aldehyde (DA) as the crosslinking agent. Gelatin nanofibrous mats were immersed in DA solution for 1, 3, and 5 days to vary the extent of crosslinking reactions. Afterwards the extent of crosslinking was gauged by exposing the crosslinked mats to water for 1 h. The results revealed that the crosslinking time of 1 day was insufficient, as it did not impart sufficient stability to the gelatin nanofibrous membranes, which became transparent and sticky after an hour of exposure to the aqueous medium. The rest of the samples with extended crosslinking reaction for 3 and 5 days remained unaffected by exposure to the same aqueous medium. In order to determine the effect of crosslinking on mechanical properties, the specimens in the form of rectangular strips having dimensions of 6 cm × 0.4 cm were cut and stretched at a crosshead speed of 10 mm/min. It was found that the crosslinking resulted in a threefold improvement in the tensile strength and tensile modulus due to covalent bonding along the nanofibers by Schiff’s base reaction with DA.

A summary of mechanical testing protocols adopted for crosslinked ENMs has been reported in Table 1.

### 4.2. Post Fabrication Drawing/Stretching of ENMs

Stretching a polymer fiber can cause molecular chains to align along the fiber axis. This essentially results in the reduction of fiber diameter, an increase in crystallinity accompanied by improvement in tensile strength and modulus and a drop in elongation at break as the crystalline domains represent closely packed, rigid, and immobile macromolecular chains. In case of electrospinning, some stretching is induced on the fibers as they move from the needle orifice towards the collector under the action of electrostatic force. Bending instabilities in this region as a result of repulsion between the bends of advancing fiber cause whipping action which stretches the polymer jet to many times its initial length. Afterwards, the collection modes, and hence the draw ratios, can be varied by changing collector configuration. This involves use of static or dynamic collectors. An example of the first case is where random fibers are deposited on a stationary collector plate connected to the ground. Alternatively, electrospun fibers can be collected on a rotating drum collector, whose surface speed determines the stretching treatment that the fibers are subjected to. This type of fiber collection mechanism also ensures fiber alignment in the direction of drum rotation. The web of fibers thus collected is anisotropic in terms of its macromechanical properties. The surface speed of the rotating collector can be thus directly correlated to the crystallinity and mechanical properties of the wound electrospun fibrous mat. Another important variation of this concept is related to the fabrication of tows or strands of electrospun fibers which are twisted and stretched at the same time before their collection on a drum or roller. While this collection mechanism also draws the fibers depending on the speed of collection device, the twist imparted during the process also enhances the mechanical integrity of the fibrous strands. The methodology can produce useful nanofiber-based yarns or tows not only for use in composite materials, but also as precursor materials for the production of high performance tows by employing further treatments and modification techniques.

Liu et al. [90] have described a novel approach of stretching a bundle of electrospun PAN nanofibers. They collected the jets of electrospun fibers emanating from three parallel spinnerets arranged in a row, on an aluminum plate submerged in water bath. The aluminum plate was electrically grounded. The deposited fibers were collected on the surface of flowing water and were then guided onto the surface of a roller with a diameter of 25 cm and rotating at a speed of 130 rpm. In order to compare the modified collection mechanism with a traditional rolling drum collector, a batch of nanofibers was also collected on a drum with a diameter of 25 cm and rotating at 200 rpm. In order to stretch these nanofibers, a rectangular frame of 10 cm × 20 cm inner dimensions was used, one end of which was tied to a metal hook and the other end was attached to a tensioning device. The two sides of the frame were cut, and the fibers were submerged in water at 97 °C for stretching. It was reported that the novel flowing water assisted collection methodology resulted in higher uniaxial alignment, and the collected bundles were found to be uniform and smooth. After stretching the nanofibers to 4 times their initial length, their diameter was found to reduce by 56%. The reduced diameter generally improves the mechanical strength, as the probability of encountering a flaw in a given length is reduced even though mechanical testing results were not presented. The crystallinity also improved by 72% as a result of the reported stretching treatment.

**Figure 6:**
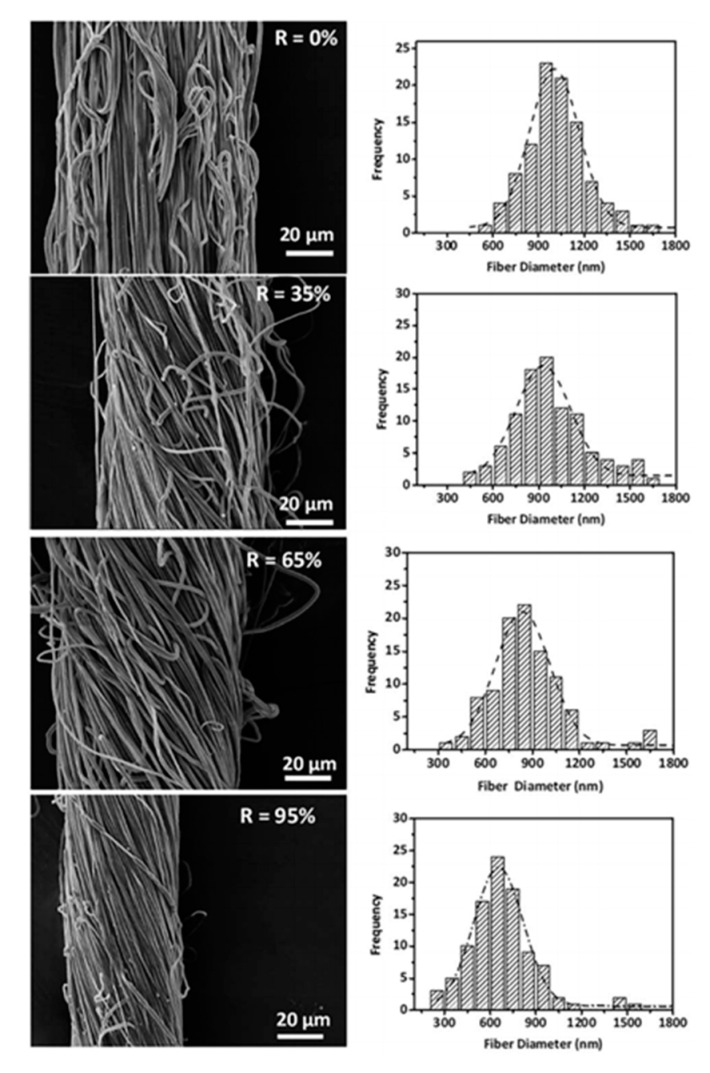
SEM (scanning electron microscope) images of nanofiber yarns and histograms of individual fiber diameter into the respective yarn produced under the stretching ratio of R = 0%, 35%, 65%, and 95% under the same twisting condition (twist multiplier = 11.5) [91] (reprinted with permission from Royal Society of Chemistry).

Ali and co-workers [91] used a rotary metal tube collector in conjunction with two needle based electrospinning nozzles, a DC power supply, and a pair of stretching rollers to simultaneously twist and stretch the nanofiber bundles in order to convert them into yarns. Yarn take up speed could be adjusted from 0.01–10 m/min, whereas the twist compensator was capable of inserting twist up to 10,000 turns per minute. Yarn surfaces also became smoother with the stretching treatment, as shown in Figure 6.

**Figure 7:**
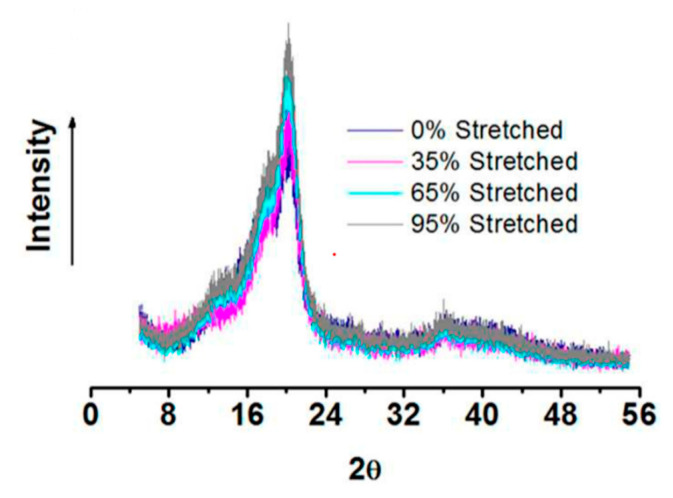
XRD (X-ray diffraction) patterns of nanofiber yarns with different stretch ratios [91] (reprinted with permission from Royal Society of Chemistry).

X-ray diffraction (XRD) results revealed that the diffraction peak intensities increased progressively with the increasing stretch ratios as is obvious from Figure 7. It was also found that the crystallite size increased as the yarn was stretched by 0%, 35%, 65%, and 95% owing to an enhanced macromolecular chain alignment along the fiber axis, along with its elongation.

**Figure 8:**
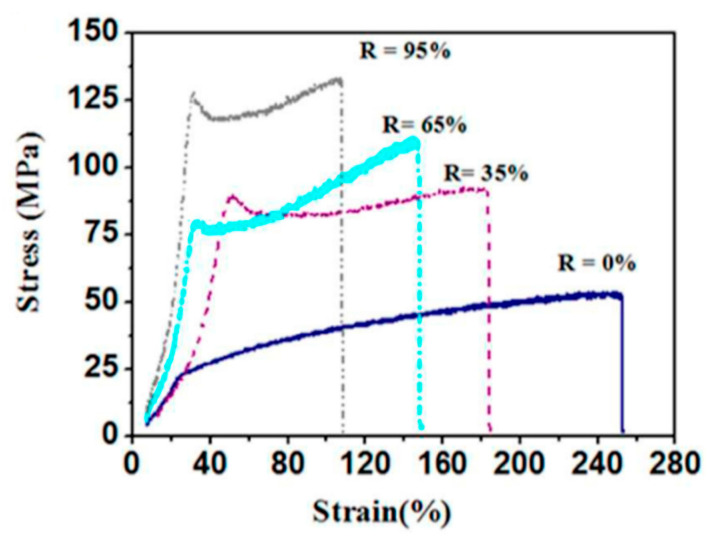
Stress–strain curves of nanofiber yarns with different stretching ratios [91] (reprinted with permission from Royal Society of Chemistry).

Tensile tests were performed on a gauge length of 30 mm at a cross head speed of 300 mm/min. The results are given in Figure 8. For stretching treatment from 0% to 95%, tensile strength increased from ~48 MPa to ~128 MPa, and Young’s modulus improved from ~60 MPa to ~334 MPa. Elongation at break decreased from ~263% to ~110%. Fiber diameters reduced from 998 ± 141 nm to 631 ± 98 nm due to stretching to 95% of the original yarn length.

Kim [92] described a novel collector head capable of orienting electrospun nanofibers in different directions. This collector head was used to obtain parallel and perpendicular orientations of aligned polycaprolactone (PCL) nanofibers. This was achieved through the use of a mobile collector moving first in the x-direction and then in the y-direction. The moving head was CAD (Computer Aided Design) operated and moved at two different speeds of 25 cm/s and 35 cm/s, representing lower and higher draw ratios, respectively. In this way, the cross over points had cross-ply orientation of nanofibers. Tensile tests on 2 mm × 15 mm specimens were carried out at a crosshead speed of 0.1 mm/s to determine the effect of the collector speed on the nanofibrous membrane properties in parallel and perpendicular directions. It was found that the Young’s modulus increased from ~24 MPa to ~27 MPa, and tensile strength improved from ~3.2 MPa to ~3.6 MPa, in the direction of deposited nanofibers, as the collector speed increased from 25 cm/s to 35 cm/s. Mechanical properties in the transverse direction remained almost unchanged.

A summary of mechanical testing protocols adopted for stretched/drawn ENMs has been reported in Table 2.

### 4.3. Solvent Welding of ENMs

Solvent welding is another technique which can be employed to selectively fuse the electrospun fibers at inter-fiber junctions to improve the mechanical integrity of the electrospun fibrous membrane. This can be done by using a single solvent or a solvent/nonsolvent mixture. The targeted polymer should be soluble in the selected solvent. From the point of view of thermodynamics, the solubility of a polymer in a solvent can be predicted by Hildebrand solubility parameter (δ) [94]. If the Hildebrand solubility parameters for solvent and the polymer are denoted as δ_s_ and δ_p_, respectively, then the criterion for solubility is mathematically stated as |δ_s_ − δ_p_| ≤ 2.

Once a suitable solvent has been selected, other factors which can be manipulated to effectively weld the fibrous membrane include the volume fraction of the solvent in the solvent/nonsolvent mixture (welding solution), temperature of the welding solution to fine tune the vapor pressure, and the time of exposure.

Halim et al. [95,96] reported a simple approach of solvent vapor welding for electrospun Nylon 6.6 membranes intended for anti-fouling filtration membranes. Their methodology was comprised of exposing the Nylon 6.6 membranes to formic acid vapor at room temperature inside a vacuum chamber for 5, 12, 24, and 48 h, respectively. The exposure window, ranging from 5–12 h, had no effect on the membrane morphology. Beyond this, for an exposure of 24 h, fiber swelling was observed. For extended exposure spanning 48 h, fibers completely swelled and fused, resulting in the loss of fibrous morphology. Surface roughness analysis carried out using AFM revealed that roughness decreased from Ra of 155.6 nm for pristine membranes to 80.63 nm after vapor exposure for 24 h. Fiber fusion resulted in reduced permeability and porosity as well.

**Figure 9:**
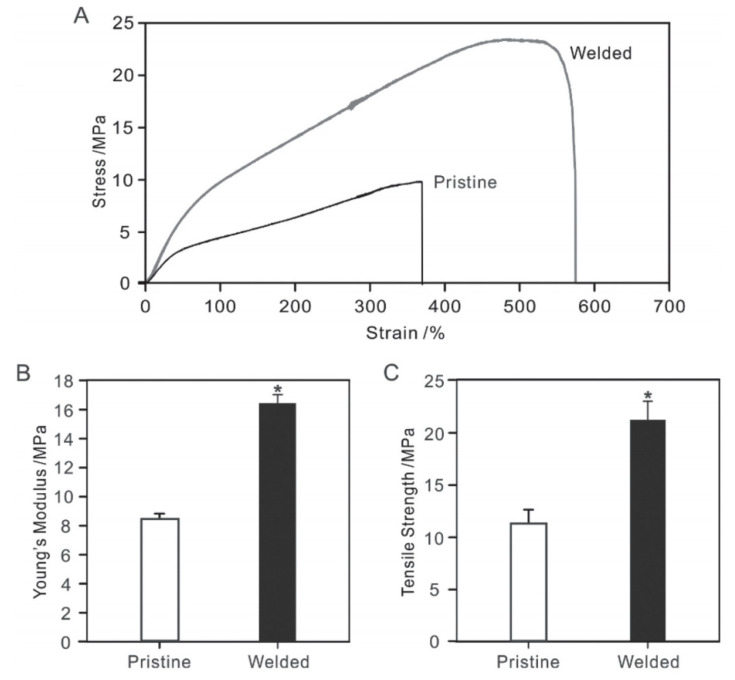
Tensile mechanical assessment of electrospun PCL nanofiber mats before and after the treatment with 25 µL dichloromethane (DCM) vapor for 60 min. (**A**) Stress–strain curves, (**B**) Young’s modulus, and (**C**) tensile strength, *n* = 5 for each test, * indicates significant difference between the two types of samples (*p* < 0.05) [97] (reprinted with permission from John Wiley & Sons).

A similar approach of solvent welding was proposed by Li et al. [97]. It was hypothesized by the authors that the exposure time and partial pressure of the solvent vapor are two critical parameters for control of the degree of swelling and the extent of welding in electrospun nanofibrous membranes. Semi-crystalline polycaprolactone (PCL) membrane specimens were exposed to dichloromethane (DCM) vapors in a closed vial. Virtually no swelling or welding was observed for 20 and 25 μL of DCM (partial pressure of DCM = 31.6 and 39.5 kPa, respectively). Evidence of fiber welding was found when the DCM volume was increased to 30 μL (Partial pressure of DCM = 47.5 kPa). Further increase in the solvent partial pressure resulted in the complete loss of fiber morphology. The effect of exposure time on welding morphology was also studied by varying exposure duration for fixed DCM volume of 25 μL. For an exposure time of 30 min at this volume, welding could be observed at the crossover points, which became even more prominent after 60 min. The welding of cross over points resulted in local alteration of morphologies, but overall fiber structure remained unaltered after the solvent vapor induced welding, as attested by the fact that the degree of crystallinity of the pristine and welded nanofibers remained the same. Fiber diameter and pore size distribution also remained largely unaffected by the reported welding procedure, as confirmed by SEM analysis and porometry. An analysis of mechanical properties revealed that the Young’s modulus and tensile strength increased twofold due to welding at cross-over points, which essentially prevents inter-fiber slippage and enhances the mechanical integrity of membranes (Figure 9A,C).

Su et al. [98] reported polyvinylidenefluoride-co-hexafluoropropylene (PVDF-HFP) electrospun nanofibrous membranes for membrane distillation (MD) applications. Membrane specimens were exposed to *N*, *N*-dimethylacetamide (DMAC) vapors in a sealed culture dish, which was heated to 65 °C for 20, 40, and 60 min separately to assess the effect of varying vapor exposure duration. The pristine nanofibrous membrane lacked structural integrity due to the absence of inter-fiber bonding at the junction points. After vapor exposure for 20 min, some bonding could be observed due to fiber fusion at cross over points. Most of the fiber junctions appeared to be welded for an exposure of 40 min beyond which entire fiber lengths started showing signs of inter-fiber fusion, resulting in loss of fiber morphology. Tensile testing carried out on pristine and welded membrane specimens revealed that the Young’s modulus, failure strain, and tensile strength increased by 117%, 79%, and 90%, respectively, after DMAC vapor exposure for 40 min, whereas an improvement of 149%, 105%, and 213%, respectively, was observed in these three parameters for an exposure spanning 60 min as inter-fiber junctions got extensively bonded for longer exposures.

In order to improve the structural integrity of polyacrylonitrile (PAN) and polysulfone (PS) membranes intended for water filtration applications, Huang and coworkers [99] enclosed their 8 cm X 8 cm small coupons in a glass dessicater saturated with DMF vapors. Two vapor exposure schemes were devised; for method A, the coupons were left on the aluminum foil to serve as the impermeable substrate, while for method B, the coupons were removed from the foil. PAN was given an exposure of 6, 9, and 18 h, whereas PS was exposed for 1, 3, and 6 h. The coupons were subsequently dried in a fume hood. For PAN membranes subjected to an exposure of 18 h using method A, the tensile strength and Young’s Modulus increased by 300% and 800%, respectively, as compared to pristine membranes. Similar exposure conditions resulted in only 56% and 25% increase in tensile strength and Young’s modulus, respectively, of PAN membranes when method B was employed. For method A treated PS membranes, the tensile strength and Young’s modulus increased fourfold after 6 h of exposure, whereas these properties only increased by 80% and 110% after the same exposure duration for method B. The use of aluminum foil as an impermeable substrate facilitated condensation of the solvent and subsequent swelling and fusion of fibers to effect welding at cross over points causing a reduction in pore size. Method A treated membranes also demonstrated improved failure strain owing to the plasticization effect of the solvent.

In certain cases, solvents can be diluted with non-solvents in order to control the extent of welding and fiber dissolution on vapor exposure. Employing this approach, cellulose acetate/polyvinyledene difluoride (CA/PVDF) nanofibrous membranes were welded using a mixture of acetone and *N*,*N*-dimethylacetamide (DMAc) [100]. The low volatility of DMAc allowed the reduction of solvent vapor pressure, which in turn helped avoid complete dissolution of fibers. The acetone/DMAc ratios employed were 1/0, 2/1, 1/2, and 0/1. The simple solvent welding approach consisted of exposing the membranes to vapors by placing them over the mouths of glass bottles filled with solvent mixtures and covering the membrane surfaces with cling wrap. The solvent exposure time was varied between 5–90 min at 50 °C. Exposure to pure acetone resulted in excessive fusion and binding of fibers, whereas pure DMAc had no significant impact on fiber morphology. The intermediate acetone/DMAc mixtures helped regulate the vapor pressure to achieve a range of morphological properties and resulting porosity of membranes through the control of weld density at fiber junctions.

Instead of introducing a welding solvent separately after the fabrication of electrospun membranes, Yoon et al. [101] used a mixture of Dimethylformamide (DMF) and *N*-methyl-pyrrolidinone (NMP) as latent solvents during electrospinning of polyethersulfone (PES) membranes. Four different ratios of NMP in the solvent mixture were employed, i.e., 25, 40, 50, and 75 wt.%, while the concentration of PES was maintained at 26 wt.%. Slow evaporation of a high boiling point solvent component (NMP) resulted in residual solvent content in the nanofibrous membranes, which resulted in their post fabrication fusion and consequent welding of fibers. For a high NMP concentration of 75%, an excessive fusion of fibers resulted in the loss of fiber morphology. Mixing of the two solvents helped in controlling the extent of inter-fiber bonding in the membranes. This was accompanied by other morphological changes in the fibers, which could be directly correlated to the solvent contents. As the NMP concentration in solvent mixture increased to 50 wt.%, fiber diameter increased from 550 nm to 760 nm. It was conjectured by the authors that this was partly because of an increase in the viscosity of the solution and partly due to a decrease in the vapor pressure of the solvent system. The tensile strength and Young’s Modulus of the membranes observed for 50/50 DMF/NMP solvent mixture, improved 360% and 570%, respectively, over the membranes obtained from PES/DMF solution. Whereas, maximum failure strain was registered for 25 wt.% NMP. The improvement in mechanical properties was attributed to inter-fiber bonding at fiber junctions, which was due to the slow rate of evaporation of NMP. NMP was found to remain ‘latent’ in the membranes after electrospinning, making itself available for inter fiber fusion after electrospinning, thus improving membrane integrity. Higher residual NMP concentrations also allowed plasticization of the fibers, thus improving the ultimate failure strain, as was the case with the 75/25 DMF/NMP solvent mixture until the inter-fiber welding restrained the movement of the fibers to offset the impact of plasticization (as observed in the 50/50 DMF/NMP solvent mixture).

Jie et al. [102] reported a solvent soaking treatment of bamboo cellulose derived cellulose acetate (B-CA) electrospun nanofibers. The membrane specimens were hung in an enclosed glass container filled with the mixture of ethanol and acetone in different volume ratios (100/0, 95/5, 90/10, and 85/15). This was followed by air drying of the specimens to remove extra solvent. The absorption of solvent was reported to induce swelling followed by condensation of the solvent at inter fiber junctions causing their local dissolution and eventual bonding. The maximum improvement in tensile strength from ~4 MPa to ~8MPa, in Young’s Modulus from ~220 MPa to ~300 MPa, and in the failure strain from ~1.8% to ~2.8% was observed in membranes treated with a 95/5 (*v*/*v*) ethanol/acetone solvent mixture. The improvement in tensile strength and modulus was attributed to optimum fusion and inter-fiber welding at junction points. The failure strain improved due to plasticization effect of the solvent.

In order to improve both the mechanical integrity and hydrophilicity of poly(vinylidene fluoride) (PVDF) electrospun membranes, solutions comprising of a nonionic surfactant, SPAN-80 in different concentrations ranging from 1, 3, 5, and 10 g/L in n-hexane were employed by Ding and coworkers [103]. Since the static immersion of the membrane resulted in an asymmetric structure of the membrane with only the top layer getting effectively welded, the welding solution was filtered through the membrane specimens, under the effect of gravity for 0.5–3 h. This helped achieve symmetric welding results through the thickness welding of fiber joints. As SPAN-80 has both hydrophilic and hydrophobic segments, the hydrophobic end attaches itself with the PVDF fiber. This helps promote fiber welding and interconnection with the PVDF fibers. The hydrophilic segment helps reduce surface tension and render the fibrous mat hydrophilic. It was also reported that an increase in surfactant content improved inter fiber bonding, especially for concentrations above 5 g/L, while inter-fiber adhesion and associated lower porosity and higher mechanical properties were observed. The impact on mechanical properties was determined on rectangular specimens having dimensions of 10 mm × 100–140 mm over a gauge length of 10 mm by conducting tensile tests at a crosshead speed of 50 mm/minute. The tensile strength was found to increase from ~1.7 MPa to ~8.8 MPa for specimens subjected to filtration assisted symmetric welding treatment for 3 h. Young’s modulus also increased from 5.5 MPa for the pristine membrane to 8.9 MPa for these membranes. Maximum strain at failure was also reported to improve as it increased from ~48% to ~80% owing to the unique surfactant assisted welding approach.

In one of the process improvements, polyvinylidene fluoride-co-hexafluoropropylene (PVDF-HFP) and PAN were welded by electrospraying the membrane with solvent (Dimethylacetamide: DMAc) and non-solvent (ethanol) mixture [104]. The effect of DMAc concentration was evaluated by varying the volume concentration of DMAc in ethanol. For 3% volume fraction DMAc, slight fusion of fibers was observed, for 5% volume fraction, most of the inter fiber junctions were found to be welded, whereas a higher volume fraction of 8% resulted in excessive welding, causing pore blockage and rendering the membrane unsuitable for filtration and distillation applications. In contrast, treatment with pure ethanol did not affect welding at any of the inter fiber junctions. The effect of temperature on solvent induced welding revealed that temperature directly influences the dissolution of fibers at inter-fiber junctions. Based on these results, a DMAc volume fraction of 5% was used to weld the membrane at 65 °C for a duration of 2 min. Fiber size distribution, porosity, and crystallinity remained unaffected by solvent vapor induced welding. Improved membrane integrity was manifested by higher tensile strength, which increased from 4.1 MPa to 8.6 MPa, increased in Young’s Modulus from 3.33 MPa to 7.78 MPa, and higher elongation at break, which increased from 84% to 134% after the solvent induced welding.

Namsaeng et al. [105] reported the strategy for welding electrospun PAN-PVC (7-1 wt.%, 6-2 wt.%, 5-3 wt.%, 4-4 wt.%) blended nanofibers made with DMF (dimethylformamide) as solvent. The polymer blend was also doped with 1 wt.%, 2.5 wt.%, 5 wt.%, and 7.5 wt.% Multi-wall carbon nanotubes (MWCNTs) in order to make composite membranes. The membrane specimens were exposed to DMF vapor in a glass dessicator for various durations, i.e., 6, 9, 15, 24, and 30 h followed by drying for 24 h. A solvent exposure time of 24 h was found to be optimal for inducing inter-fiber fusion at fiber junctions, as shorter exposure times did not cause significant welding due to small quantities of condensed solvent available at the fiber crossover points. Longer duration of 30 h caused excessive welding, which reduced membrane porosity. Once exposure duration was optimized in terms of welding efficiency, the membranes were tested in tensile mode at a crosshead speed of 10 mm/min. The gauge length was maintained at 50 mm while the specimen dimensions were 70 mm × 10 mm. In line with previous findings, tensile strength and modulus improved by 127% and 175%, respectively, due to welding at fiber junctions, but maximum tensile elongation at break reduced from 12% to 7%. The act of doping the polymer blend with 1 wt.% MWCNTs was found to improve the tensile strength and modulus by a further 205% and 314%, respectively.

A summary of mechanical testing protocols adopted for solvent welded ENMs has been reported in Table 3.

### 4.4. Heat Treatment/Annealing of ENMs

Heat treatment of electrospun fibrous membranes is another technique of interest for the enhancement of their mechanical properties. Heat treatment or annealing of these membranes/fibrous webs results in improved crystallinity due to the rearrangement of polymer chains at higher temperatures. In addition to that, partial fiber fusion may also occur, leading to inter-fiber welding at fiber crossover points, which gives these membranes structural integrity. For optimum results and in order to avoid complete melting of fibrous membrane, it was suggested that annealing should be carried out above crystallization temperature and below melting point of the polymer [72]. Moreover, duration of annealing should be long enough to allow rearrangement of all the chains. Heating temperature and duration are thus two factors which can be manipulated to tailor the properties of the heat treated electrospun membrane. Heat treatment in these conditions results in improved crystallinity due to rearrangement of molecular chains in the amorphous regions in addition to inter fiber fusion due to partial melting of fibers at junction points. In many cases these two factors, i.e., enhanced crystallinity and welding of fibers at crossover points contribute to improve mechanical properties of the electrospun fibrous membranes.

Tan et al. [72] reported annealing of electrospun poly (L-lactic acid) (PLLA) nanofibers. Annealing was conducted at 75 °C for 24 h, a temperature close to the crystallization temperature which helps avert melting of low molecular weight polymer chains. Moreover, the long duration of annealing ensured that all the polymer chains had enough time to rearrange themselves. It was found that annealing turned amorphous regions into denser crystallites. Interfibrillar regions are the ones with the least stiffness. Annealing was found to result in the formation of crystallites in these interfibrillar regions, which in turn increases the resistance of the fiber to deformation. When load is applied along the fiber axis, the interfibrillar tie molecules are stretched. More than twofold improvement in Young’s modulus was observed as a result of annealing, as it increased from ~4.7 GPa to ~11.3 GPa. Annealing also caused reduction in diameter by 10%.

Another strategy of selecting the annealing temperature based on the same principle was adopted by Mahir and coworkers [106]. The temperature selection principle is based on the premise that higher post treatment temperatures involve more melting and recrystallization, which render the material brittle. The PVA membranes were thus heat treated at *Tg* (85 °C = ~0.5 of the melting temperature, *Tm*) and ~1.65 *Tg* (140 °C = ~0.7 *Tm*). In this way, the selected temperatures were well below the decomposition temperature of the polymer. It was observed that the heat treatment caused partial merging owing to fusion of fibers, the extent of which increased with temperature. The careful selection of heat treatment temperatures resulted in improvement in mechanical properties as revealed by tensile tests. For pristine samples, the tensile yield stress was 2.4–6.98 MPa, Young’s Modulus was 103–128 KPa, and average elongation at break was found to be from 35.02–59.81%. For the specimens heat treated at 85 °C and 140 °C, the yield stress improved to 3.63–9.63 MPa and 4.11–6.3 MPa, the improved average Young’s Modulus was 110–137 KPa and 109–137 KPa, while average elongation at break reduced to 28.82–31.26% and 21.47–29.71%, respectively.

Tissue engineering scaffolds were electrospun using 10% chitosan blended with 20% gelatin (CG) in 90% acetic acid [107]. The CG membranes were annealed in a vacuum oven at four different annealing temperatures, i.e., 60, 90, 120, and 150 °C. The thermal treatment was carried out for 90 min followed by cooling of the specimens to room temperature. Annealing at 60 °C produced only 15% and 8% improvement, while 90 °C resulted in 1.3-fold and 1.1-fold increases in Young’s Modulus and tensile strength, respectively. Membranes annealed at 60 °C demonstrated high ductility, whereas CG membranes which were annealed above *Tg* (90, 120, and 150 °C) exhibit ductile-to-brittle transition as depicted by brittle fracture and absence of yielding region. Tensile strength and Young’s modulus attained the maximum value for heat treatment at 150 °C. Elongation at break on the other hand decreased with the increase in annealing temperature. Energy to break was maximum for the specimens heat treated at 90 °C due to a combined effect of relatively high tensile strength and Young’s modulus. Annealed and unannealed specimens had similar diffraction patterns indicating that the extent of crystallinity remained unaffected by annealing. High tensile strength and Young’s modulus together with low elongation at break for annealed specimens could thus be attributed to inter-fiber welding due to thermal treatment.

Polyvinylidene fluoride (PVDF) membranes were heat treated at three different temperatures (150, 155, and 160 °C) in vacuum for two hours. Contrary to other approaches presented earlier, the three heat treatment temperatures were close to the melting point of the polymer in powdered form (Tm of PVDF = 159.5 °C).

Increasing heat treatment temperature resulted in an increase in fiber diameter and a wider distribution. DSC (Differential Scanning Calorimetry) and WAXD (Wide-angle X-ray scattering) revealed that crystallinity first increased for heat treatment temperatures of 150 °C and 155 °C and decreased by heat treating at 160 °C. It has been conjectured by the authors that this is due to the formation of unstable secondary crystallites at heat treatment temperatures of 150 °C and 155 °C resulting in an increase in melting enthalpy and crystallinity, which eventually decrease for membranes heat treated at 160 °C due to melting of these crystallites. These results are summarized in Table 4.

**Figure 10:**
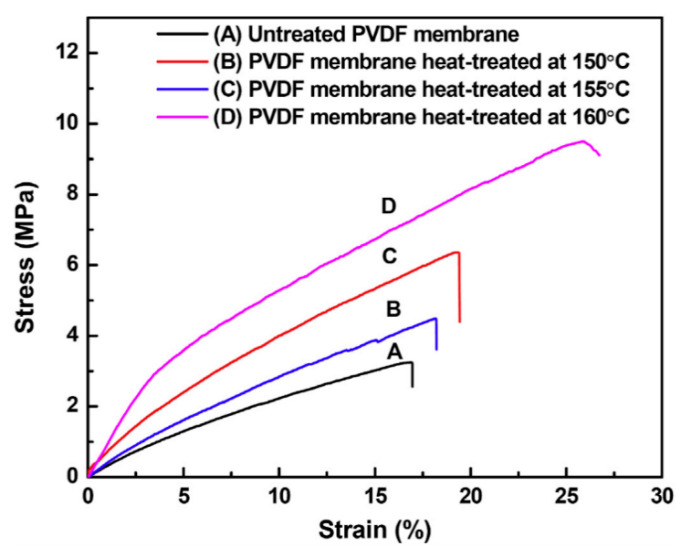
Stress–strain curves of PVDF fibrous membranes before and after heat treatment at different temperatures [108] (reprinted with permission from Elsevier).

The pristine and heat-treated membranes were loaded in tensile mode over a gauge length of 30 mm at a crosshead speed of 5 mm/min. It was reported that the combined effect of an increase in fiber diameter, bonding of fibers due to local fusion, and enhanced crystallinity improved not only the tensile strength and modulus, but elongation at break as well. Tensile strength, Young’s modulus, and elongation at break% were 3.25 MPa, 38.9 MPa, and 16.9%, respectively, for untreated membranes. These three improved to 4.49 MPa, 48.1 MPa, and 18.2% for heat treatment conducted at 150 °C, 6.36 MPa, 84.7 MPa, and 19.4% for heat treatment conducted at 155 °C, and finally to 9.50 MPa, 94.2 MPa and 26.7% for heat treatment conducted at 160 °C. These results are given in Figure 10.

A broad range of temperatures were employed by Ramaswamy et al. [109] for thermal treatment of a composite polymer solution of poly(L-lactic) acid doped with multi-walled carbon nanotubes (0.25, 0.5, 1, 2, 3 wt.%). Temperatures selected were just above *Tg* (70 and 80 °C), between glass transition *Tg* and melting point, *Tm* (120 °C), close to melting temperature, *Tm* (150, 160, and 170 °C). Heat treatment was carried out in a convection oven for five minutes. In order to gauge the effect of annealing temperature on the mechanical properties of membranes, tensile tests were carried out at 10 mm/min over a gauge length of 30 mm. It was found that thermal treatment temperature close to the melting point resulted in the loss of fibrous morphology and a film like structure was obtained, but with significantly higher crystallinity resulting in the highest strength and modulus. Notwithstanding these results, the highest heat treatment temperature that preserves the fibrous morphology should be selected for heat treatment. The heat treatment temperature of 120 °C, which lies between *Tg* and *Tm*, resulted in inter-fiber fusion and helped maximize tensile strength. Based on the obtained results, it was suggested that in order to achieve high modulus, a treatment temperature close to *Tg* should be maintained, which allows inter-fiber bonding without the relaxation of polymer chains.

A slightly different approach of heat treating vascular scaffolds was reported by Lee et al. [110]. Electrospun poly(3-caprolactone) (PCL) scaffolds were treated with Pluronic F127, a lower critical solution temperature (LCST) polymer which gels in distilled water at 20 °C. Vacuum assisted in the penetration of the gel in the scaffold pores. Pluronic F127 improves dimensional stability of the fibrous scaffold during its subsequent thermal treatment and helps avert shrinkage when fibers are being fused together for welding. The scaffolds were then placed in a warm water bath to heat treat at temperatures ranging from 54 °C to 60 °C as the PCL scaffolds were found to melt at 61.9 °C. Afterwards, the scaffolds were washed with distilled water at 4 °C for 48 h. Heat treatment temperatures were selected to lie between *Tg* and *Tm* for optimum results. In order to evaluate the effect of heat treatment on the mechanical integrity of scaffolds, tensile tests were conducted at a crosshead speed of 8 mm/min on 10 mm × 5 mm × 0.3 mm specimens. The tensile strength for scaffold specimens treated at 55 °C was found to be 9.1 MPa against 5.1 MPa for the pristine ones. Similarly, elongation at break for scaffolds treated at 55 °C was 675%, whereas the untreated specimens demonstrated a value of 417%. The improvement in tensile properties was attributed to inter fiber bonding in the scaffolds.

Polyacrylonitrile (PAN)/fluorinated polyurethane (FPU) nanofibrous composite membranes for waterproof breathable structures were reported by Sheng and coworkers [111]. After the initial trials with various polymer concentrations, it was found that 8 wt.% PAN and 10 wt.% FPU gave the optimum bead free fiber morphologies. The authors have also reported a straight forward empirical approach to the selection of post fabrication heat treatment temperature. The composite membranes were first heat treated at 100 °C, which is slightly above the glass transition temperature. Since fiber bonding was not observed after the treatment at this temperature, the membranes were subsequently subjected to higher heat treatment temperatures of 120 °C, 140 °C, and 160 °C, which lie between the glass transition temperature and the melting point. At these temperatures, polymer chains were more mobile and were able to diffuse into the neighboring fibers at the junction points. Thus, increasing temperature helped improve inter-fiber bonding. Molecular rearrangement also caused the fiber diameter to decrease as the heat treatment temperature increased. Maximum pore size also decreased, whereas porosity increased. The former could be attributed to a decreasing trend of fiber diameters, while the latter is due to decreasing tortuosity of the pores when they are welded together. Tensile tests revealed that the pristine membranes had a nonintegrated structure with a low tensile strength of 3.11 MPa and elongation at break% age of 37.6%. Membranes heated at 140 °C exhibited maximum enhancement over the untreated membranes with a tensile strength of 9.4 MPa, representing a threefold increase. This was accompanied by a decrease in elongation at break to ~32%. The increase in tensile strength and reduction in elongation at break is attributed to a change in failure mode as slippage at fiber crossover points is the dominant failure mode in untreated membranes, since they are merely held by frictional contact between the fibers. This gives way to fiber failure and junction breakage in heat treated membranes when subjected to stretching forces as the fiber crossover points are welded together due to polymer fusion.

An interesting improvement of the original thermal treatment process is comprised of the use of two polymers; one of which is high melting point and the other one is low melting point. The thermal treatment can be carried out at the temperature where the low melting point polymer would melt and fuse the high melting point fibers together. In this way, a ‘matrix’ of molten polymer can be used to achieve inter-fiber bonding of ‘reinforcing fibers’. In order to demonstrate this concept, different architectures of PCL and PLA nanofibers were fabricated by Kancheva et al. [112]. In this combination, PLA has a high melting point of 165 °C, whereas PCL melts at 60 °C. One of the architectures was made by blending PCL and PLA polymers in different wt/wt ratios, i.e., 75/25, 60/40, and 50/50. These blends were used to deposit electrospun membranes from a single solution ejected from a solitary electrospinning nozzle. Sandwich type mats were prepared by electrospinning the PCL membrane and then PLA over it and vice versa. The two variants were called PCL+PLA and PLA + PCL, respectively. The third architecture was made by simultaneous electrospinning of PLA and PCL solutions by using two separate needles working side by side. In order to fabricate the fourth architecture, simultaneous electrospinning of PLA solution and electrospraying of PCL solution was employed using two nozzles. All of the architectures were heat treated at 60 °C for 15 min, which allowed the melting of PCL and therefore fusion of PLA fibers at crossover points. Blended architectures gave the greatest enhancement in mechanical properties. Sandwich type architectures had the PLA nanofibers completely bonded by the surrounding molten ‘matrix’ of PCL nanofibers. PCL nanofibers or particles fused to enhance mechanical integrity of membranes in the other two types of architectures as well.

A summary of mechanical testing protocols adopted for annealed ENMs has been reported in Table 5.

### 4.5. Hot Pressing of ENMs

Simultaneous application of heat and pressure is another approach that has been explored by various researchers in order to enhance the mechanical integrity of membranes. Temperature and pressure are two fundamental parameters which need to be worked with in order to optimize hot pressing treatment results. Hot pressing generally results in the increase in fiber diameter due to heat assisted compaction of the fibrous web resulting in inter-fiber welding and resultant structural integrity.

**Figure 11:**
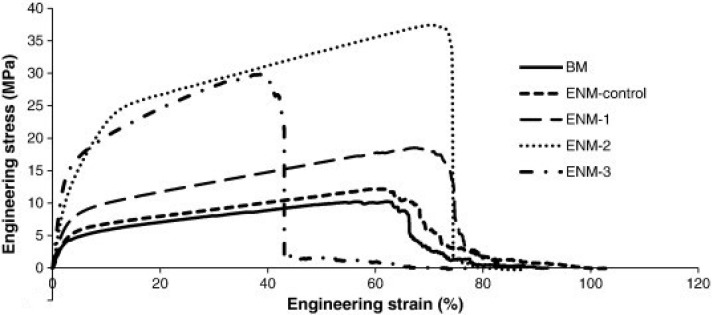
Mechanical properties of the various ENMs and nonwoven backing material (BM) [113] (reprinted with permission from Elsevier).

Kaur et al. [113] have reported polyacrylonitrile (PAN) membrane electrospun on the surface of nonwoven polyester backing material intended for application in nanofiltration membranes. The ENM specimens comprising of a support layer and deposited PAN fibers were first treated with mild convective air in a fume hood for around 3 h in order to remove residual solvent. A thermal transfer press was then used to hot press the ENM at 87 °C for 999 s. Three different pressures were applied simultaneously, ranging from 0.14 MPa, 0.28 MPa, to 0.41 MPa. For 0.14 MPa pressure, no significant change in fiber diameters was found. Fiber diameters, however increased for higher applied pressures during heat treatment. This was accompanied by the fusion of multiple fibers and their bonding. Thickness of ENMs decreased with increasing pressure. Mechanical properties were also reported to improve with hot pressing owing to increase in fiber diameter and crystallization (refer to Figure 11). Pristine ENM specimens (ENM-control in Figure 11) were found to have 5.7 MPa and 9.8 MPa yield strength and tensile strength, respectively. For ENMs treated at 0.14 MPa (ENM-1 in Figure 11), yield stress and tensile strength were improved to 6.2 MPa and 13.93 MPa, respectively, which represented 9% and 4% improvement. For the ENMs treated at 0.28 MPa (ENM-2 in Figure 11), yield stress and tensile strength improved by 313% and 203%, respectively, when compared with the pristine specimens. For applied pressure of 0.41 MPa (ENM-3 in Figure 11), the yield stress and tensile strength reduced by 26% and 19% as compared to specimens heat treated at 0.28 MPa.

Improvement of the mechanical properties of electrospun membranes is of the utmost importance for their application as lithium-ion battery separators. Gong et al. [114] electrospun poly(phthalazinone) ether sulfone ketone (PPESK) membranes in both random and aligned configurations. The aligned ones were then laid over one another in cross ply fashion. These membranes were first vacuum dried at 120 °C for 24 h in order to remove residual solvent, which was followed by hot pressing at 320 °C under 2 MPa applied pressure.

In order to gauge the impact of the hot pressing treatment on the mechanical properties of ENMs, specimens of 10 mm × 50 mm were tested at a crosshead speed of 5 mm/min under tensile loading mode. For membranes having random orientation of fibers, the tensile strength was found to be 1.7 MPa. After deposition of oriented fibers on the collecting drum, the tensile strength in the direction of fibers was 16.4 MPa (tensile strength in the transverse directions was less than 1 MPa). When cross ply nanofibrous membrane was hot pressed, the tensile strength improved significantly and reached 22.8 MPa in both the directions. Morphological analysis revealed that the hot pressed nanofibers had larger diameters as compared to nanofibers in pristine membranes due to the hot pressing treatment.

PVDF membranes have also been used extensively for filtration and battery separation. In a slight variation to the static hot pressing, hot press for continuous heat treatment of PVDF membranes can also be employed [115]. The PVDF electrospun membrane was fed into the continuous hot presser at 135 °C, running at 0.43 m/min. Since PVDF melts at 160–166 °C, the treatment temperature was chosen to preserve the fibrous morphology of membranes. In order to ensure homogenous heat treatment, both upper and lower surfaces were treated by feeding the membrane twice in the press. Inter fiber bonding, especially at the surfaces, improved integrity of the membranes even though a comparison of the mechanical properties of heat treated membranes with the pristine ones was not given.

A similar group has reported monolayer and double layer PVDF electrospun membranes hot pressed in a continuous manner at 25–155 °C [116]. Tensile tests were conducted to gauge the impact of hot pressing on the mechanical properties. It was revealed that the Young’s modulus, tensile strength, and elongation at break improved after hot pressing treatment. For hot pressing treatment at 130 °C, the monolayer and double layer membranes showed a rise of tensile moduli from ~18 MPa to ~200 MPa and ~170 MPa, respectively. Tensile modulus increased to even higher values after heat treatment at 145 °C and 155 °C, as Young’s modulus was reported to be 602 MPa for hot pressing treatment at 155 °C. Nevertheless, the temperatures above 130 °C were not suitable for the intended application, as porosity decreased at higher temperatures. Relaxation of internal stresses and crystalline perfection resulted in the improvement in tensile strength which rose from ~2 MPa to ~21 MPa and ~19 MPa for monolayer and double layer membranes, respectively. Maximum elongation at break of 88% was observed for double layer membranes hot pressed at 130 °C. At higher temperatures, the elongation at break% decreased significantly and was only 8% after hot pressing at 155 °C. Hot pressing resulted in reduction in membrane thickness and porosity as well. SEM analysis revealed that for hot pressing treatments up till 130 °C, fiber morphology was also found to remain intact. At higher temperatures (145 °C and 155 °C) melting and resultant changes in fiber morphology were observed. At these temperatures recrystallization of the polymer nanofibers also caused formation of α-type crystalline form as revealed by XRD results. The polymeric fibers however, did not show an increase in crystallinity due to hot pressing.

Given the importance of Li ion battery separators and advantages associated with the use of electrospun nanofibrous membranes, some researchers have reported copolymers of poly(vinylidene) fluoride such as polyvinylidenefluoride-co-hexafluoropropylene (PVDF-HFP) [117]. These copolymer membranes were hot pressed at 70 °C in order to enhance their mechanical integrity. In one such interesting demonstration, PVDF-HFP electrospun membranes were hot pressed by passing a heated household iron for 1–2 s over the membrane sandwiched between aluminum foil and papers to avoid direct contact of the membrane surface with the heated iron [118]. The surface temperature of the iron was kept at ~200 °C, which was above the melting point of the polymer (~150 °C). After removal of heat treated membranes, they were folded such that the bottom surfaces were in contact. In order to simulate the actual pressure that is required to rupture the membrane, a Mullen burst test was performed. Pristine as well as single layer and double layer sandwiched membranes were tested for the determination of burst pressure. It was found that an increase in polymer concentration, which results in higher fiber diameters, gives high burst pressure results. Hot pressing treatment also improves the mechanical properties and burst resistance, as does the increase in thickness of the membrane by sandwiching technique as the highest burst pressure was found to be 28.2 PSI for the double layer sandwiched membranes.

A summary of mechanical testing protocols adopted for hot pressed ENMs has been reported in Table 6.

### 4.6. Hot Stretching of ENMs

Hot stretching carried out above glass transition temperature results in the reduction of fiber diameters accompanied by an increase in the crystallinity owing to macromolecular reorientation along the fiber axis. This is accompanied by welding at cross over points. All of these factors contribute towards enhanced structural integrity. Under the influence of a tensile load, the randomly arranged fibers first align along the direction of load and then stretch when inter-fiber locking no longer allows slippage of fibers. Temperatures in excess of glass transition are generally recommended, as the chain mobility above glass transition temperature facilitates macromolecular reorientation along the fiber axes. A higher temperature would allow stretching treatment to be carried out at a relatively small tensile load. Applied tensile load and temperature are thus dependent on the polymer type and fiber alignment in the electrospun membrane.

To demonstrate the effectiveness of hot stretching treatment, poly(m-phenylene) isophthalamide (PMIA) nanofiber membrane specimens (80 mm × 40 mm) were affixed to a specially designed fixture for use inside an air oven [119]. The membrane was heated at 270 °C, which was selected to be higher than its *Tg* (160.9 °C). Tensile loads of 6, 8, 10, and 12 N were applied for 15 min each, in order to stretch the membranes. It was observed that the diameter of nanofibers reduced as a result of hot stretching treatment. X-ray diffraction analysis of pristine and hot stretched membranes revealed that the diffraction peak location did not change before and after the treatment, indicating that the crystalline structure of nanofibers remained unaffected by the treatment. However, increased chain mobility around glass transition allowed enhanced macromolecular orientation in the amorphous region, resulting in an increase in the crystallinity revealed by increased diffraction peak intensity and sharpness. Another important transformation which accompanies hot stretching is related to fiber alignment along the loading direction, which increases anisotropy and hence mechanical properties in the fiber direction. These two factors (macromolecular chain orientation and fiber orientation), due to hot stretching, resulted in an increase in tensile strength and modulus while at the same time inter-fiber bonding and greater molecular chain and nanofiber orientation caused a reduction in elongation at break. It was reported that the highest improvement in mechanical strength and modulus (50% and 196%, respectively) were observed when hot stretching treatment involved 12 N force.

Polysulfone (PS) fibrous membranes were electrospun and subsequently heat treated in relaxed and stretched states [120]. For relaxed heating, the membrane specimens were hung in an oven prior to heating. For tension heating, two 50 g clamps were attached to the opposite ends of horizontally arranged membrane specimens deposited on aluminum foil. Fiber diameters did not change when relaxed heating mode was applied whereas the tension heating mode generally resulted in a decreasing trend in diameter with the increasing temperature. Maximum improvement in mechanical properties was observed when the specimens were hot stretched at 190 °C for 3 h, as the Young’s modulus increased from ~35 MPa for pristine membrane to ~84 MPa, tensile strength increased from ~0.67 MPa for pristine membrane to ~4.96 MPa, and failure strain increased from 25.33% for pristine membrane to 104%. It was also reported that hot stretching against relaxed mode heating resulted in greater membrane surface smoothness and dimensional stability.

Given their importance as precursors for the fabrication of high-performance carbon fibers, PAN has been extensively investigated to study the impact of hot stretching on crystallinity and mechanical properties. A self-bundling electrospinning technique was employed by Wang and co-workers to manufacture polyacrylonitrile (PAN) tows [121]. PAN nanofiber strands or tows were drawn by fixing across a clamp to various draw ratios at a constant rate (100 mm/min) in a water bath at 95 °C. Annealing of the stretched tows was carried out at 130 °C in an oven for 1 h. This temperature lies between its glass transition temperature (87 °C) and degradation temperature, which was found to lie between the range; 180–240 °C [122]. During traditional electrospinning process, jet whipping before its collection on the plate or collection roller causes its stretching and hence a reduction in fiber diameter. It was observed that the average fiber diameter of PAN fibers in the tows was slightly larger than in randomly deposited membranes due to restricted jet whipping in self-bundling technique. The crystallinity increased after drawing and annealing treatment from 16.93% to 49.20% due to macromolecular arrangement induced by stretching and subsequent heating. As the polymer chains get more aligned along the fiber axis with increased draw ratios, a direct relationship between the drawing treatment and mechanical properties was found. The highest tensile strength of 372 MPa was reported for 300% draw ratio, representing a more than 8-fold improvement over the tensile strength of pristine PAN tows (45 MPa). Similarly, the highest Young’s modulus was reported for 300% drawn strand as it increased from 0.8 GPa for untreated tows to 11.8 GPa for hot stretched ones. As expected, elongation at break followed a downward trend as it decreased from 88.6% for untreated tows to 12% for 300% drawn and annealed strands.

Song et al. [123] fabricated electrospun PAN nanofibers and their composite counterpart by doping PAN with single walled carbon nanotubes (SWNTs) in different proportions (0.25 wt.%, 5 wt.%, 0.75 wt.% and 1 wt.%) for use as carbon nanofiber precursors. PAN and PAN/SWNT membrane specimens were affixed with graphite plates which hung in an oven. Each membrane specimen was stretched using 75 g weight, which was suspended from the free end of the specimen. The stretching treatment was applied at 135 °C for 5 min. X-ray diffraction analysis of pristine and hot stretched nanofibers revealed that the untreated nanofibers had limited crystallinity (one weak diffraction peak with 2θ value of 17.0°) while hot stretched nanofibers were identified by two sharp and intense diffraction peaks with 2θ values of 17.0° and 29.5°. The resultant enhancement in percent crystallinity was threefold. It was also reported that orientation factor (f) increased from 0.22 to 0.76 after hot stretching. Crystallite size was also reported to increase by ~162%. These results attest to a higher degree of macromolecular orientation and order along the fiber axis, leading to improved mechanical properties. Tensile tests on pristine and hot stretched membrane specimens revealed that tensile strength and modulus increased by 55.32% and 156.48%, respectively, for PAN nanofibers and by 54.70% and 125.40%, respectively, for PAN/SWNT nanofibers. Similar PAN/SWNT composite nanofibrous membranes were reported by Hou et al. [124]. An identical hot stretching methodology resulted in threefold improvement in crystallinity. As expected, greater macromolecular orientation and crystallinity resulted in increasing the crystallite size, as well as improvement in tensile strength and Young’s modulus. This was accompanied by reduction in elongation at break from ~22% to ~12%. As reported by Wu et al. [125], the aforementioned hot stretching protocol resulted in their PAN membranes stretching by a factor of 1.7. Hot stretching caused four-fold improvement in the degree of crystallinity, 22% increase in orientation factor, and a 10% reduction in crystallite size. This was accompanied by an increase in tensile strength and Young’s modulus by a factor of ~4.7 and ~5.7, respectively. The failure strain at the same time was reduced by 50%.

A similar approach of stretching PAN electrospun nanofibers was implemented by suspending the nanofiber membranes in an oven at ~100 °C [126]. The membranes were hot stretched to 2, 3, and 4 times their original length with the help of a stretching device immersed in a boiling water bath. As previously reported, hot stretching was found to reduce fiber diameter. A direct relationship was found between the hot stretch ratio and macromolecular orientation, as well as polymer crystallinity. It was reported that crystallinity and Herman’s orientation function reached maximum values of 72.9 % and 0.94, respectively, for membranes stretched to 4 times their original length. The treatment also improved the tensile strength and Young’s modulus by 333% and 413%, respectively, as tensile strength increased from ~15 MPa to ~65 MPa and Young’s modulus improved from ~275 MPa to ~1412 MPa.

**Figure 12:**
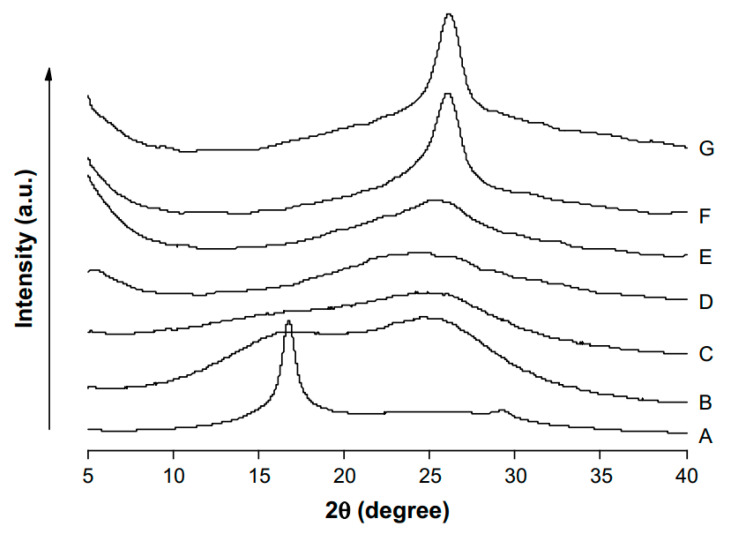
XRD curves of (**A**) as-electrospun PAN nanofibers, (**B**) stabilized commercial PAN (SAF 3K) precursor fibers, (**C**) stabilized PAN nanofibers, (**D**) 1000 °C carbonized PAN nanofibers, (**E**) 1400 °C carbonized PAN nanofibers, (**F**) 1800 °C carbonized PAN nanofibers, and (**G**) 2200 °C carbonized PAN nanofibers [127] (reprinted with permission from Elsevier).

Zhou et al. [127] have reported the complete manufacturing route of carbon nanofibers from electrospun PAN nanofibers. Once the PAN nanofibers were electrospun, these were removed from the collector and wrapped around a glass rod with a certain degree of tension. This was followed by stabilization of the PAN nanofiber bundles at 280 °C for 3 h. The stabilization process crosslinks the polymer and creates a ladder like structure which is thermally stable and does not melt during subsequent processing. Stabilization was followed by the traditional processing step of carbonization at 1000 °C in an inert atmosphere. Subsequently, graphitization was carried out at higher temperatures of 1400 °C, 1800 °C, and 2200 °C for 1 h. Morphological analysis revealed that even though average fiber diameter after stabilization was the same as for precursor PAN nanofibers, it was reduced after carbonization and high temperature graphitization treatments. After carbonization treatment, the carbonaceous structure achieved was turbostratic with folded carbon sheets arranged in haphazard fashion. After graphitization treatment, the graphitic structure was obtained with graphene sheets arranged in a ribbon-like configuration. Higher graphitic content achieved at high temperature treatments can also be discerned from progressively increasing intensity and sharpness of peaks for membrane specimens treated at 1400 °C, 1800 °C, and 2200 °C, respectively, as shown in Figure 12.

**Figure 13:**
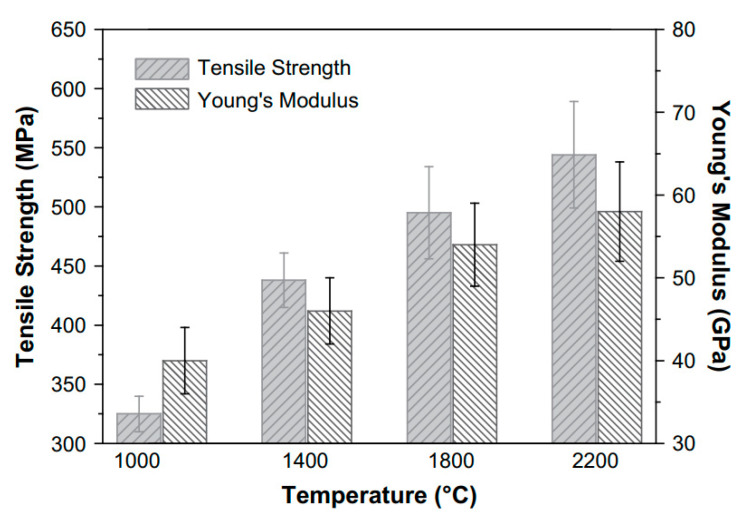
Tensile strength and Young’s moduli of electrospun carbon nanofiber bundles [127] (reprinted with permission from Elsevier).

The tensile strength and Young’s modulus for PAN nanofibers carbonized at 1000 °C were ~325 MPa and ~40 GPa, respectively. After graphitization treatment at 2200 °C, the tensile strength and Young’s modulus improved to ~542 MPa and ~58 GPa, respectively, representing ~67% and ~45% improvement in the two parameters. Figure 13 gives a comparative analysis of tensile strength and Young’s modulus for membranes subjected to carbonization and graphitization treatments at different temperatures.

A summary of mechanical testing protocols adopted for hot stretched ENMs has been reported in Table 7.

## 5. Discussion of Key Findings

Electrospinning is an attractive fiber fabrication technique specifically for the synthesis of nanoscale fibers and their membranes due to its simplicity and freedom to use a broad spectrum of polymeric systems. A range of parameters related to process, materials, environment and collector configuration have to be optimized in order to achieve successful electrospinning of a given polymer. Once this is done, these same parameters can be varied within the electrospinning window to alter the various properties of electrospun fibers including mechanical properties which have been the focus of this review paper.

The effect of the variation of these parameters and their optimization can be gauged through the execution of mechanical tests. These tests can either be conducted on single fibers using advanced testing systems and protocols or alternatively on the membrane specimens which are in the form of web of interconnected fibers. The former needs special equipment and single fiber handling expertise, which are costly and time-consuming undertakings. The latter scheme is not only simpler and less costly, but can also give useful information about the effectiveness of the optimization scheme undertaken. The importance of such tests is twofold, as electrospun membranes often need post fabrication treatment and determination of the effectiveness of these treatments in relatively short spans of time is essential.

It has been observed that electrospun fibers collected in the form of an interconnected web or membrane either in random or aligned orientation lack structural coherence and strength needed for their effective exploitation in various application areas. This is so even though these membranes undergo an optimization at the manufacturing stage through the variation/alteration of various parameters as described earlier. A post fabrication strength enhancement strategy is therefore indispensable for all practical exploitation of electrospun fibrous membranes.

This literature survey has revealed a myriad of techniques available for the post fabrication strength enhancement of nanofibrous membranes. These include chemical as well as physical methods which can be adopted and tailored according to the requirements of the electrospun membrane in question.

Techniques like chemical crosslinking, thermal annealing, solvent dissolution, and stretching/drawing treatments are unassisted, as the first one is purely chemical, while the other three are solely physical. On the other hand, there are so called ‘assisted techniques’, such as hot pressing and hot stretching, which are thermally assisted mechanical treatment methods. These latter two lead to greater recrystallization and associated molecular rearrangement in the amorphous domains as well as fiber welding at inter fiber junctions, giving rise to higher crystallinity, alteration in fiber diameter, and improvement in structural integrity.

Table 8 gives a brief overview of various post fabrication treatments discussed in this review.

These changes work in tandem to improve mechanical properties such as tensile strength and modulus, while altering failure strain due to improved structural integrity in the membranes, which are among the most studied and compared parameters. At the same time, these post fabrication treatments should preserve fibrous morphology of the membranes.

Optimization of the properties during the execution of one of these chemical or physical schemes is carried out by carefully controlling the parameters and avoiding over exposure, which might result in the loss of fibrous morphology. Improvement in mechanical properties at the loss of fibrous morphology is avoided, as it results in simultaneous reduction in porosity and tortuosity in membranes. At the same time, inherent advantages associated with the use of ultrafine/nano fibers are also sacrificed. This is also one of the challenges in the selection and effective execution of one of the post fabrication strength enhancement techniques.

Certain generalized trends can be identified in the literature which help understand the effect of post fabrication treatments on morphological and mechanical properties of electrospun fibers. These findings have been summarized in Table 9.

A detailed discussion of testing protocols adopted in the literature for the comparison of untreated and treated membranes has also been provided. A uniform testing standard is far from available, and no uniform testing methodology has been adopted so far. Nevertheless, certain trends can be discerned, including a preference for uniaxial tensile testing on membrane specimens for a quick and meaningful comparative analysis of the effectiveness of the treatment scheme adopted. Single fiber testing protocols have rarely been adopted due to complexities and costs involved. A standardized testing procedure to bridge the gap between single fiber and membrane testing protocols while clearly establishing a link between the two is needed.

## 6. Conclusions

This review discusses in detail the control of key parameters during electrospinning which affect the mechanical properties of membranes, various testing protocols, and procedures which can be adopted to assess mechanical strength of the membranes followed by the post-processing strategies for the enhancement of mechanical properties of ENMs. It is expected that this review will enable more innovative research on post processing strategies of ENMs by providing thorough understanding of the comparative merits of these techniques for a particular electrospun fibrous membrane. An optimized treatment methodology may also be devised by hybridizing one or several of the schemes for greater crystallinity and enhanced mechanical properties while preserving the fibrous morphologies.

## Figures and Tables

**Table 1:** Testing protocols adopted for ENMs—crosslinked.

ENM	Load Cell	Crosshead Speed	Specimen Dim.	Gauge Length	Remarks	Ref.
Chitosan (CTS)	50 N	30 mm/min	5 cm × 1.0 cm	Not specified	Uniaxial tensile test	[83]
Poly(vinyl alcohol) (PVA)	Not specified	2 mm/min	20 mm × 5 mm × 100µm (length × width × thickness)	10 mm	Uniaxial tensile test	[84]
Gelatin	100 N	10 mm/min	6 cm × 0.4 cm × 0.2 mm	Not specified	Uniaxial tensile test	[88]
Polyacrylonitrile (PAN) and crosslinked polyvinyl alcohol (PVA)	Not specified	10 mm/min	Dumbbell shaped specimens, Narrow width at center: 4 mm. Specimen thicknesses: 70–90 µm	40 mm	Uniaxial tensile test (standard: DIN53504-S3A)	[85]
Polymers of Intrinsic Microporosity (HPIM)/Polybenzoxazine (BA-a)	Not specified	Force ramp rate: 0.05 N/min	8 mm × 6 mm × 0.1 mm	Not specified	Dynamic mechanical analysis (DMA)	[87]
Sodium alginate (SA) and pullulan (PUL)	5 kg	5 mm/s	30 × 10 mm	20 mm	Uniaxial tensile test	[89]

**Table 2:** Testing protocols adopted for ENMs—stretched/drawn.

ENM	Load Cell	Crosshead Speed	Specimen Dim.	Gauge Length	Remarks	Ref.
Polyacrylonitrile (PCL)	Not specified	0.1 mm/s	2 mm × 15 mm	Not specified	Uniaxial tensile test	[92]
Polyacrylonitrile (PAN)	Not specified	2 mm/min	Not specified	20 mm	Uniaxial tensile test	[93]
Poly(vinylidene fluoride-co-hexafluoropropylene) (PVDF-HFP)	Not specified	300 mm/min	Not specified	30 mm	Uniaxial tensile test	[91]

**Table 3:** Testing protocols adopted for ENMs—solvent weld.

ENM	Load Cell	Crosshead Speed	Specimen Dim.	Gauge Length	Remarks	Ref.
Polyvinylidene fluoride-co-hexafluoropropylene (PVDF-HFP) And polyacrylonitrile (PAN)	Not specified	3 mm/s	Not specified	Not specified	Electronic fabric strength tester was used	[104]
Polyvinylidene fluoride-co-hexafluoropropylene (PVDF-HFP)	Not specified	3 mm/s	Not specified	Not specified	Electronic fabric strength tester was used	[98]
Polyethersulfone (PES)	Not specified	10 mm/min	Width at the center of the specimen: 4 mm, specimen thickness: ~40 μm	25 mm	Uniaxial tensile test	[101]
Polyacylonitrile (PAN) and polysulfone (PSu)	Not specified	Not specified	40 mm × 5.5 mm	Not specified	Dynamic Mechanical Analysis	[99]
Polyvinylidene fluoride (PVDF)	Not specified	10 mm/min	Width: 10 mm Length: 100–140 mm	50 mm	Uniaxial tensile test	[103]
Polyacrylonitrile-poly(vinyl chloride) (PAN–PVC)	Not specified	10 mm/min	70 mm × 10 mm, Thickness of as-spun mat: ~0.7 mm, Thickness of post-treated mats ~0.2 mm	50 mm	Uniaxial tensile test	[105]

**Table 4:** Thermal properties and degrees of crystallinity of polyvinylidene fluoride (PVDF) fibrous membranes [108] (reprinted with permission from Elsevier).

Sample No.	Melting Temperature (°C)	Melting Enthalpy (Jg^−1^)	Crystallinity (%)	
			DSC	WAXD
PVDF powder	159.5	23.4	22.3	-
A (untreated)	161.4	35.7	34.1	42.1
B (Heat-treated at 150 °C)	161.7	45.4	43.3	53.5
C (Heat-treated at 155 °C)	162.7	53.0	50.6	58.1
D (Heat-treated at 160 °C)	166.3	36.1	34.6	33.00

**Table 5:** Testing protocols adopted for ENMs–annealed.

ENM	Load Cell	Crosshead Speed	Specimen Dim.	Gauge Length	Remarks	Ref.
Poly (L-lactic acid) (PLLA)	Not applicable	Not applicable	Not applicable	Not applicable	Single fiber three-point bend test, AFM cantilever spring constant: 0.15 N/m Loading rate: 5 µm/s Maximum load: 9 nN. Nanofibers were deposited on silicon wafer having micro sized etched grooves	[72]
Polyvinyl alcohol (PVA)	Not specified	1, 5, 10, and 100 mm/min	Not specified	Not specified	ASTM D 882-2002 standard Uniaxial tensile test	[106]
Poly(L-lactic acid) (PLA) and poly(ε-caprolactone) (PCL)	2 mV/V, Type: Xforce P, Nominal force: 2.5 kN	20 mm/min	20 mm × 60 mm, Thickness: ca. 200 µm	Not specified	Uniaxial tensile test	[112]
Chitosan-gelatin (CG)	200 N	20 mm/min	60 mm × 5 mm	40 mm	Uniaxial tensile test	[107]
Polyvinylidene fluoride (PVDF)	Not specified	5 mm/min	Not specified	30 mm	Uniaxial tensile test	[108]
Poly lactic acid (PLA)– Multi-wall carbon nanotubes (MWCNTs)	Not specified	10 mm/min	width of 1.5 cm Thickness: 50 mm	3 cm	Uniaxial tensile test	[109]
Poly (ε-caprolactone) (PCL)	Not specified	0.5 mm/s	Tubular scaffold: Diameter: 4.75 mm, Thickness: 0.3 mm, Length: 15 mm	Not specified	Uniaxial tensile test	[110]
Polyacrylonitrile (PAN)/ fluorinated polyurethane (FPU)	200 N	20 mm/min	30 mm × 3 mm	10 mm	Uniaxial tensile test	[111]

**Table 6:** Testing protocols adopted for ENMs—hot pressed.

ENM	Load Cell	Crosshead Speed	Specimen Dim.	Gauge Length	Remarks	Ref.
Polyacrylonitrile (PAN)	Not specified	Not specified	8 cm × 2 cm	Not specified	Uniaxial tensile test	[113]
Poly(phthalazinone ether sulfone ketone) (PPESK)	Not specified	5 mm/min	10 mm × 50 mm	30 mm	Uniaxial tensile test	[114]
Polyvinylidene fluoride (PVDF)	100 N	5 mm/min	60 mm × 10 mm	40 mm	Uniaxial tensile test	[115]
Poly(vinylidene fluoride (PVDF)	Not specified	5 mm/min	60 mm × 10 mm	40 mm	Uniaxial tensile test	[116]
Polyvinylidenefluoride-co-hexafluoropropylene (PVDF-HFP)	Not applicable	Not applicable	50 mm × 50 mm	Not applicable	Mullen-burst test	[118]

**Table 7:** Testing protocols adopted for ENMs—hot stretched.

ENM	Load Cell	Crosshead Speed	Specimen Dim.	Gauge Length	Remarks	Ref.
Polyacrylonitrile (PAN)	Not specified	0.5 mm/min	1.5 cm × 1.5 cm (Cardboard frame outer dimensions)	1 cm × 1 cm (Cardboard frame inner dimensions)	Uniaxial tensile test	[127]
Poly(mphenylene) isophthalamide Nanofibers (PMIA)	Not specified	20 mm/min	50 mm × 5 mm	20 mm	Uniaxial tensile test	[119]
Polysulfone (PSU)	Measuring accuracy of strength: 0.01 cN, Measuring accuracy of elongation: 0.01 mm	10 mm/min	40 mm × 2 mm	10 mm	Uniaxial tensile test	[120]
Polyacrylonitrile (PAN)	Not specified	15 mm/min	Not specified	30 mm	Uniaxial tensile test	[121]
Polyacrylonitrile (PAN)	Not specified	20 mm/min	20 mm × 5 mm	Not specified	Uniaxial tensile test	[123]
Polyacrylonitrile (PAN)	Force resolution of 50 μN	0.05 mm/min	10 mm	Not specified	Uniaxial tensile test	[126]
Polyacrylonitrile (PAN)	Not specified	For dried electrospun fibrous mats: 10 mm/min Dried, twisted yarns of electrospun nanofibers: 2 mm/min	Not specified	20 mm	For electrospun mats: ASTM 1708D Uniaxial tensile test	[128]
Polyacrylonitrile (PAN)	Not specified	Not specified	20 mm × 5 mm	Not specified	Uniaxial tensile test	[124]
Polyacrylonitrile (PAN)	Not specified	Not specified	20 mm × 5 mm	Not specified	Uniaxial tensile test	[125]

**Table 8:** Overview of post fabrication strategies for enhancement of mechanical properties of ENMs.

Nature of Treatment	Primary Control Parameter	Secondary Control Parameter	Remarks
**Crosslinking**	Crosslinker concentration	Temperature	Crosslinker compatibility and reactivity are critical factors.
**Annealing**	Temperature	Duration	Temperature range should be maintained above crystallization temperature (Tc) and below melting point (Tm), which usually results in increased crystallinity.
			Temperatures maintained between Tg and Tm result in better strength.
			Temperatures maintained close to Tg result in improved elastic modulus.
**Hot stretching**	Temperature and stretching force	Duration	
**Hot pressing**	Temperature and applied pressure	Duration	Temperatures should be below Tm.
			Generally, temperatures lower than those used for annealing are employed.
			If temperature is too close to the melting point, the duration of treatment should be significantly reduced to preserve fibrous morphology.
**Solvent welding**	Vapor pressure	Duration	Solubility of polymer in the solvent is an important determinant, the criterion for which is:
			|δ_s_ − δ_p_| ≤ 2 *.
**Stretching/drawing**	Draw ratio	None	In certain cases, an optional twist may also be applied.

* Hildebrand solubility parameter for solvent and the polymer are denoted as δ_s_ and δ_p_, respectively [94].

**Table 9:** Summary of the effect of post fabrication treatments on the properties of ENMs.

Nature of Treatment	Effect of the Treatment on					Remarks
	Diameter	Crystallinity	Tensile Strength	Young’s Modulus	Failure Strain	
**Crosslinking**	No significant impact	Decreases	Increases	Increases	Usually decreases	Crosslinker concentration has significant impact on the overall properties.
**Annealing**	Depends on the type of polymer and annealing temperature selected (if fibers undergo melting as a result of heat treatment, diameter increases)	Usually Increases (when treatment carried out above crystallization temperature)	Increases	Increases	Depends on the type of polymer and annealing temperature selected	A range of temperatures selected between Tg and Tm or Tc and Tm can have diversely different effects on the properties of different polymers. If treatment is carried out at T > Tc, crystallinity increases.
**Hot** **stretching**	Decreases	Increases	Increases	Increases	Depends on the type of polymer and hot pressing conditions	
**Hot** **pressing**	Increases	Increases	Increases	Increases	Depends on the type of polymer and hot pressing conditions	
**Solvent** **welding**	Unaffected	Unaffected	Increases	Increases	Usually increases	Failure strain increases when plasticization effect of the solvent dominates.
**Stretching/** **drawing**	Decreases	Increases	Increases	Increases	Decreases	

## References

[1] Unique Properties (2016). Essentials in Nanoscience and Nanotechnology.

[2] Sudha P.N., Sangeetha K., Vijayalakshmi K., Barhoum A., Barhoum A., Makhlouf A.S.H. (2018). Chapter 12—Nanomaterials history, classification, unique properties, production and market. Emerging Applications of Nanoparticles and Architecture Nanostructures.

[3] Adekoya J.A., Ogunniran K.O., Siyanbola T.O., Dare E.O., Revaprasadu N., Seehra M., Bristow A.D. (2018). Band structure, morphology, functionality, and size-dependent properties of metal nanoparticles. Noble and Precious Metals—Properties, Nanoscale Effects and Applications.

[4] Madkour L.H. (2019). Processing of Nanomaterials (NMs). Nanoelectronic Materials.

[5] Rabiee M., Rabiee N., Salarian R., Rabiee G. (2019). Nanomaterials: Concepts. Introduction to Nanomaterials in Medicine.

[6] Behera A., Mohapatra S.S., Verma D.K. (2019). Nanomaterials: Fundamental Principle and Applications. Nanotechnology and Nanomaterial Applications in Food, Health, and Biomedical Sciences.

[7] Naz M.Y., Shukrullah S., Ghaffar A., Ali K., Sharma S. (2020). Synthesis and Processing of Nanomaterials. Solar Cells.

[8] Taylor G.I. (1964). Disintegration of water drops in an electric field. Proc. R. Soc. Lond. Ser. A. Math. Phys. Sci..

[9] Haider A., Haider S., Kang I.-K. (2018). A comprehensive review summarizing the effect of electrospinning parameters and potential applications of nanofibers in biomedical and biotechnology. Arab. J. Chem..

[10] Huang Z.-M., Zhang Y.-Z., Kotaki M., Ramakrishna S. (2003). A review on polymer nanofibers by electrospinning and their applications in nanocomposites. Compos. Sci. Technol..

[11] Basson N. (2014). Free Volume of Electrospun Organic-Inorganic Copolymers.

[12] Nezafati N., Faridi-Majidi R., Pazouki M., Hesaraki S. (2019). Synthesis and characterization of a novel freeze-dried silanated chitosan bone tissue engineering scaffold reinforced with electrospun hydroxyapatite nanofiber. Polym. Int..

[13] Vazquez J.J., Martínez E.S.M. (2019). Collagen and elastin scaffold by electrospinning for skin tissue engineering applications. J. Mater. Res..

[14] Heidari M., Bahrami S.H., Ranjbar-Mohammadi M., Milan P. (2019). Smart electrospun nanofibers containing PCL/gelatin/graphene oxide for application in nerve tissue engineering. Mater. Sci. Eng. C.

[15] Liu X., Yang Y., Yu D.-G., Zhu M.-J., Zhao M., Williams G.R. (2019). Tunable zero-order drug delivery systems created by modified triaxial electrospinning. Chem. Eng. J..

[16] Ding Y., Li W., Zhang F., Liu Z., Zanjanizadeh Ezazi N., Liu D., Santos H.A. (2019). Electrospun fibrous architectures for drug delivery, tissue engineering and cancer therapy. Adv. Funct. Mater..

[17] Topuz F., Uyar T. (2019). Electrospinning of cyclodextrin functional nanofibers for drug delivery applications. Pharmaceutics.

[18] Wang R., Wang T. (2019). Immobilization of enzymes into nanofiber membranes via electrospinning and its application in natural sweetener production. FASEB J..

[19] Cloete W.J., Hayward S., Swart P., Klumperman B. (2019). Degradation of proteins and starch by combined immobilization of protease, α-amylase and β-galactosidase on a single electrospun nanofibrous membrane. Molecules.

[20] Jun S.-H., Yang J., Jeon H., Kim H.S., Pack S.P., Jin E., Kim J. (2020). Stabilized and Immobilized Carbonic Anhydrase on Electrospun Nanofibers for Enzymatic CO_2_ Conversion and Utilization in Expedited Microalgal Growth. Environ. Sci. Technol..

[21] Wutticharoenmongkol P., Hannirojram P., Nuthong P. (2019). Gallic acid-loaded electrospun cellulose acetate nanofibers as potential wound dressing materials. Polym. Adv. Technol..

[22] Adeli H., Khorasani M.T., Parvazinia M. (2019). Wound dressing based on electrospun PVA/chitosan/starch nanofibrous mats: Fabrication, antibacterial and cytocompatibility evaluation and in vitro healing assay. Int. J. Biol. Macromol..

[23] Yang J., Wang K., Yu D.-G., Yang Y., Bligh S.W.A., Williams G.R. (2020). Electrospun Janus nanofibers loaded with a drug and inorganic nanoparticles as an effective antibacterial wound dressing. Mater. Sci. Eng. C.

[24] Zhu M., Xiong R., Huang C. (2019). Bio-based and photocrosslinked electrospun antibacterial nanofibrous membranes for air filtration. Carbohydr. Polym..

[25] Kang Y., Wang C., Qiao Y., Gu J., Zhang H., Peijs T., Kong J., Zhang G., Shi X. (2019). Tissue-engineered trachea consisting of electrospun patterned sc-PLA/GO-g-IL fibrous membranes with antibacterial property and 3D-printed skeletons with elasticity. Biomacromolecules.

[26] Liang M., Wang F., Liu M., Yu J., Si Y., Ding B. (2019). N-Halamine Functionalized Electrospun Poly (Vinyl Alcohol-co-Ethylene) Nanofibrous Membranes with Rechargeable Antibacterial Activity for Bioprotective Applications. Adv. Fiber Mater..

[27] Lu Y., Xiao X., Fu J., Huan C., Qi S., Zhan Y., Zhu Y., Xu G. (2019). Novel smart textile with phase change materials encapsulated core-sheath structure fabricated by coaxial electrospinning. Chem. Eng. J..

[28] Lu Y., Xiao X., Liu Y., Wang J., Qi S., Huan C., Liu H., Zhu Y., Xu G. (2020). Achieving multifunctional smart textile with long afterglow and thermo-regulation via coaxial electrospinning. J. Alloy. Compd..

[29] Ciera L., Beladjal L., Van Landuyt L., Menger D., Holdinga M., Mertens J., Van Langenhove L., De Clerk K., Gheysens T. (2019). Electrospinning repellents in polyvinyl alcohol-nanofibres for obtaining mosquito-repelling fabrics. R. Soc. Open Sci..

[30] Chen Y., Qiu L., Ma X., Chu Z., Zhuang Z., Dong L., Du P., Xiong J. (2020). Electrospun PMIA and PVDF-HFP composite nanofibrous membranes with two different structures for improved lithium-ion battery separators. Solid State Ion..

[31] Yanilmaz M. (2020). Evaluation of electrospun PVA/SiO_2_ nanofiber separator membranes for lithium-ion batteries. J. Text. Inst..

[32] Xu Y., Zhu J.-W., Fang J.-B., Li X., Yu M., Long Y.-Z. (2020). Electrospun High-Thermal-Resistant Inorganic Composite Nonwoven as Lithium-Ion Battery Separator. J. Nanomater..

[33] Gattenby C., Olarte S., Murray D. (2019). Free Surface Electrospun Polyvinylidene Fluoride Membranes for Direct Contact Membrane Distillation. http://www.electrostatics.org/images/H5.pdf.

[34] Khayet M., García-Payo C., Matsuura T. (2019). Superhydrophobic nanofibers electrospun by surface segregating fluorinated amphiphilic additive for membrane distillation. J. Membr. Sci..

[35] Li K., Hou D., Fu C., Wang K., Wang J. (2019). Fabrication of PVDF nanofibrous hydrophobic composite membranes reinforced with fabric substrates via electrospinning for membrane distillation desalination. J. Environ. Sci..

[36] Lv D., Wang R., Tang G., Mou Z., Lei J., Han J., De Smedt S., Xiong R., Huang C. (2019). Ecofriendly electrospun membranes loaded with visible-light-responding nanoparticles for multifunctional usages: Highly efficient air filtration, dye scavenging, and bactericidal activity. ACS Appl. Mater. Interfaces.

[37] Gao H., He W., Zhao Y.-B., Opris D.M., Xu G., Wang J. (2020). Electret mechanisms and kinetics of electrospun nanofiber membranes and lifetime in filtration applications in comparison with corona-charged membranes. J. Membr. Sci..

[38] Zhang S., Shi Q., Christodoulatos C., Korfiatis G., Meng X. (2019). Adsorptive filtration of lead by electrospun PVA/PAA nanofiber membranes in a fixed-bed column. Chem. Eng. J..

[39] Pan C.-T., Chang C.-C., Yang Y.-S., Yen C.-K., Kao Y.-H., Shiue Y.-L. (2020). Development of MMG sensors using PVDF piezoelectric electrospinning for lower limb rehabilitation exoskeleton. Sens. Actuators A Phys..

[40] Schoolaert E., Hoogenboom R., De Clerck K. Going from Polymer to Application: Solvent Electrospinning of Optical Nanofibrous Sensors. Proceedings of the 19th World Textile Conference (AUTEX-2019): Textiles at the Crossroads.

[41] Avossa J., Paolesse R., Di Natale C., Zampetti E., Bertoni G., De Cesare F., Scarascia-Mugnozza G., Macagnano A. (2019). Electrospinning of Polystyrene/Polyhydroxybutyrate Nanofibers Doped with Porphyrin and Graphene for Chemiresistor Gas Sensors. Nanomaterials.

[42] Pozegic T., King S., Fotouhi M., Stolojan V., Silva S., Hamerton I. (2019). Delivering interlaminar reinforcement in composites through electrospun nanofibres. Adv. Manuf. Polym. Compos. Sci..

[43] Deeraj B., Saritha A., Joseph K. (2019). Electrospun styrene-butadiene copolymer fibers as potential reinforcement in epoxy composites: Modeling of rheological and visco elastic data. Compos. Part B Eng..

[44] Shakil U.A., Hassan S.B., Yahya M.Y., Nauman S. (2020). Mechanical properties of electrospun nanofiber reinforced/interleaved epoxy matrix composites—A review. Polym. Compos..

[45] Li P., Shang Z., Cui K., Zhang H., Qiao Z., Zhu C., Zhao N., Xu J. (2019). Coaxial electrospinning core-shell fibers for self-healing scratch on coatings. Chin. Chem. Lett..

[46] Li J., Hu Y., Qiu H., Yang G., Zheng S., Yang J. (2019). Coaxial electrospun fibres with graphene oxide/PAN shells for self-healing waterborne polyurethane coatings. Prog. Org. Coat..

[47] Xu S., Li J., Qiu H., Xue Y., Yang J. (2020). Repeated self-healing of composite coatings with core-shell fibres. Compos. Commun..

[48] Beachley V., Wen X. (2009). Effect of electrospinning parameters on the nanofiber diameter and length. Mater. Sci. Eng. C.

[49] Fallahi D., Rafizadeh M., Mohammadi N., Vahidi B. (2008). Effect of applied voltage on jet electric current and flow rate in electrospinning of polyacrylonitrile solutions. Polym. Int..

[50] Yördem O.S., Papila M., Menceloğlu Y.Z. (2008). Effects of electrospinning parameters on polyacrylonitrile nanofiber diameter: An investigation by response surface methodology. Mater. Des..

[51] Khalf A., Singarapu K., Madihally S.V. (2015). Influence of solvent characteristics in triaxial electrospun fiber formation. React. Funct. Polym..

[52] He J.-H., Wan Y.-Q., Yu J.-Y. (2008). Effect of concentration on electrospun polyacrylonitrile (PAN) nanofibers. Fibers Polym..

[53] Son W.K., Youk J.H., Lee T.S., Park W.H. (2004). The effects of solution properties and polyelectrolyte on electrospinning of ultrafine poly(ethylene oxide) fibers. Polymer.

[54] Nezarati R.M., Eifert M.B., Cosgriff-Hernandez E. (2013). Effects of humidity and solution viscosity on electrospun fiber morphology. Tissue Eng. Part C Methods.

[55] De Vrieze S., Van Camp T., Nelvig A., Hagström B., Westbroek P., De Clerck K. (2009). The effect of temperature and humidity on electrospinning. J. Mater. Sci..

[56] Zhao J., Liu H., Xu L. (2016). Preparation and formation mechanism of highly aligned electrospun nanofibers using a modified parallel electrode method. Mater. Des..

[57] Katta P., Alessandro M., Ramsier R.D., Chase G.G. (2004). Continuous Electrospinning of Aligned Polymer Nanofibers onto a Wire Drum Collector. Nano Lett..

[58] Li D., Wang Y., Xia Y. (2004). Electrospinning nanofibers as uniaxially aligned arrays and layer-by-layer stacked films. Adv. Mater..

[59] Theron A., Zussman E., Yarin A. (2001). Electrostatic field-assisted alignment of electrospun nanofibres. Nanotechnology.

[60] Kameoka J., Craighead H. (2003). Fabrication of oriented polymeric nanofibers on planar surfaces by electrospinning. Appl. Phys. Lett..

[61] Teo W., Kotaki M., Mo X., Ramakrishna S. (2005). Porous tubular structures with controlled fibre orientation using a modified electrospinning method. Nanotechnology.

[62] Sundaray B., Subramanian V., Natarajan T., Xiang R.-Z., Chang C.-C., Fann W.-S. (2004). Electrospinning of continuous aligned polymer fibers. Appl. Phys. Lett..

[63] Dalton P.D., Klee D., Möller M. (2005). Electrospinning with dual collection rings. Polymer.

[64] Dos Santos A., Dierck J., Troch M., Podevijn M., Schacht E. (2011). Production of continuous electrospun mats with improved mechanical properties. Macromol. Mater. Eng..

[65] Tan E., Lim C. (2006). Mechanical Characterization of a Single Nanofiber. Nanomechanics of Materials and Structures.

[66] Tan E., Lim C. (2006). Mechanical characterization of nanofibers–A review. Compos. Sci. Technol..

[67] Hang F., Lu D., Bailey R.J., Jimenez-Palomar I., Stachewicz U., Cortes-Ballesteros B., Davies M., Zech M., Bödefeld C., Barber A.H. (2011). In situ tensile testing of nanofibers by combining atomic force microscopy and scanning electron microscopy. Nanotechnology.

[68] Tan E., Lim C. (2004). Novel approach to tensile testing of micro-and nanoscale fibers. Rev. Sci. Instrum..

[69] Baker S.R., Banerjee S., Bonin K., Guthold M. (2016). Determining the mechanical properties of electrospun poly-ε-caprolactone (PCL) nanofibers using AFM and a novel fiber anchoring technique. Mater. Sci. Eng. C.

[70] Gestos A., Whitten P.G., Spinks G.M., Wallace G.G. (2013). Tensile testing of individual glassy, rubbery and hydrogel electrospun polymer nanofibres to high strain using the atomic force microscope. Polym. Test..

[71] Bazbouz M.B., Stylios G.K. (2010). The tensile properties of electrospun nylon 6 single nanofibers. J. Polym. Sci. Part B Polym. Phys..

[72] Tan E.P., Lim C. (2006). Effects of annealing on the structural and mechanical properties of electrospun polymeric nanofibres. Nanotechnology.

[73] Croisier F., Duwez A.S., Jérôme C., Léonard A.F., van der Werf K.O., Dijkstra P.J., Bennink M.L. (2012). Mechanical testing of electrospun PCL fibers. Acta Biomater..

[74] Tan E., Lim C. (2004). Physical properties of a single polymeric nanofiber. Appl. Phys. Lett..

[75] Gu S.Y., Wu Q.L., Ren J., Vancso G.J. (2005). Mechanical properties of a single electrospun fiber and its structures. Macromol. Rapid Commun..

[76] Ganser C., Hirn U., Rohm S., Schennach R., Teichert C. (2014). AFM nanoindentation of pulp fibers and thin cellulose films at varying relative humidity. Holzforschung.

[77] Standard Test Method for Tensile Properties of Thin Plastic Sheeting. https://www.astm.org/Standards/D882.

[78] Marras S.I., Kladi K.P., Tsivintzelis I., Zuburtikudis I., Panayiotou C. (2008). Biodegradable polymer nanocomposites: The role of nanoclays on the thermomechanical characteristics and the electrospun fibrous structure. Acta Biomater..

[79] Roodbar Shojaei T., Hajalilou A., Tabatabaei M., Mobli H., Aghbashlo M., Barhoum A., Bechelany M., Makhlouf A.S.H. (2019). Characterization and Evaluation of Nanofiber Materials. Handbook of Nanofibers.

[80] Wee-Eong T. ElectrospinTech. http://electrospintech.com/SOP-ES2002.html#.XrP4_mgzYdU.

[81] Kaur S., Sundarrajan S., Rana D., Sridhar R., Gopal R., Matsuura T., Ramakrishna S. (2014). Review: The characterization of electrospun nanofibrous liquid filtration membranes. J. Mater. Sci..

[82] Lee H., Yamaguchi K., Nagaishi T., Murai M., Kim M., Wei K., Zhang K.-Q., Kim I.S. (2017). Enhancement of mechanical properties of polymeric nanofibers by controlling crystallization behavior using a simple freezing/thawing process. RSC Adv..

[83] Li Q., Wang X., Lou X., Yuan H., Tu H., Li B., Zhang Y. (2015). Genipin-crosslinked electrospun chitosan nanofibers: Determination of crosslinking conditions and evaluation of cytocompatibility. Carbohydr. Polym..

[84] Wang X., Fang D., Yoon K., Hsiao B.S., Chu B. (2006). High performance ultrafiltration composite membranes based on poly (vinyl alcohol) hydrogel coating on crosslinked nanofibrous poly (vinyl alcohol) scaffold. J. Membr. Sci..

[85] Yoon K., Hsiao B.S., Chu B. (2009). High flux ultrafiltration nanofibrous membranes based on polyacrylonitrile electrospun scaffolds and crosslinked polyvinyl alcohol coating. J. Membr. Sci..

[86] Tian M., Liao Y., Wang R. (2020). Engineering a superwetting thin film nanofibrous composite membrane with excellent antifouling and self-cleaning properties to separate surfactant-stabilized oil-in-water emulsions. J. Membr. Sci..

[87] Satilmis B., Uyar T. (2019). Fabrication of thermally crosslinked hydrolyzed polymers of intrinsic microporosity (HPIM)/polybenzoxazine electrospun nanofibrous membranes. Macromol. Chem. Phys..

[88] Jalaja K., Kumar P.A., Dey T., Kundu S.C., James N.R. (2014). Modified dextran cross-linked electrospun gelatin nanofibres for biomedical applications. Carbohydr. Polym..

[89] Malgarim Cordenonsi L., Faccendini A., Rossi S., Bonferoni M.C., Malavasi L., Raffin R., Scherman Schapoval E.E., Del Fante C., Vigani B., Miele D. (2019). Platelet lysate loaded electrospun scaffolds: Effect of nanofiber types on wound healing. Eur. J. Pharm. Biopharm..

[90] Liu J., Chen G., Gao H., Zhang L., Ma S., Liang J., Fong H. (2012). Structure and thermo-chemical properties of continuous bundles of aligned and stretched electrospun polyacrylonitrile precursor nanofibers collected in a flowing water bath. Carbon.

[91] Ali U., Niu H., Abbas A., Shao H., Lin T. (2016). Online stretching of directly electrospun nanofiber yarns. RSC Adv..

[92] Kim G.H. (2008). Electrospun PCL nanofibers with anisotropic mechanical properties as a biomedical scaffold. Biomed. Mater..

[93] Fennessey S.F., Farris R.J. (2004). Fabrication of aligned and molecularly oriented electrospun polyacrylonitrile nanofibers and the mechanical behavior of their twisted yarns. Polymer.

[94] Barton A.F.M. (1975). Solubility parameters. Chem. Rev..

[95] Halim N., Wirzal M., Bilad M., Yusoff A., Nordin N., Putra Z., Jaafar J. (2018). Effect of Solvent Vapor Treatment on Electrospun Nylon 6, 6 Nanofiber Membrane. Proceedings of IOP Conference Series: Materials Science and Engineering.

[96] Halim A., Syakinah N., Wirzal M.D.H., Bilad M.R., Nordin M., Hadi N.A., Adi Putra Z., Sambudi N.S., Yusoff M., Rahim A. (2019). Improving Performance of Electrospun Nylon 6, 6 Nanofiber Membrane for Produced Water Filtration via Solvent Vapor Treatment. Polymers.

[97] Li H., Zhu C., Xue J., Ke Q., Xia Y. (2017). Enhancing the mechanical properties of electrospun nanofiber mats through controllable welding at the cross points. Macromol. Rapid Commun..

[98] Su C., Lu C., Cao H., Tang K., Chang J., Duan F., Ma X., Li Y. (2018). Fabrication and post-treatment of nanofibers-covered hollow fiber membranes for membrane distillation. J. Membr. Sci..

[99] Huang L., Manickam S.S., McCutcheon J.R. (2013). Increasing strength of electrospun nanofiber membranes for water filtration using solvent vapor. J. Membr. Sci..

[100] Liu C., Li X., Liu T., Liu Z., Li N., Zhang Y., Xiao C., Feng X. (2016). Microporous CA/PVDF membranes based on electrospun nanofibers with controlled crosslinking induced by solvent vapor. J. Membr. Sci..

[101] Yoon K., Hsiao B.S., Chu B. (2009). Formation of functional polyethersulfone electrospun membrane for water purification by mixed solvent and oxidation processes. Polymer.

[102] Cai J., Zhang Q., Lei M., He J., Liu G. (2016). The use of solvent-soaking treatment to enhance the anisotropic mechanical properties of electrospun nanofiber membranes for water filtration. RSC Adv..

[103] Ding Y., Wu J., Wang J., Lin H., Wang J., Liu G., Pei X., Liu F., Tang C.Y. (2019). Superhydrophilic and mechanical robust PVDF nanofibrous membrane through facile interfacial SPAN-80 welding for excellent oil/water separation. Appl. Surf. Sci..

[104] Su C., Lu C., Horseman T., Cao H., Duan F., Li L., Li M., Li Y. (2020). Dilute solvent welding: A quick and scalable approach for enhancing the mechanical properties and narrowing the pore size distribution of electrospun nanofibrous membrane. J. Membr. Sci..

[105] Namsaeng J., Punyodom W., Worajittiphon P. (2019). Synergistic effect of welding electrospun fibers and MWCNT reinforcement on strength enhancement of PAN–PVC non-woven mats for water filtration. Chem. Eng. Sci..

[106] Es-Saheb M., Elzatahry A. (2014). Post-heat treatment and mechanical assessment of polyvinyl alcohol nanofiber sheet fabricated by electrospinning technique. Int. J. Polym. Sci..

[107] Wang Z., Cai N., Dai Q., Li C., Hou D., Luo X., Xue Y., Yu F. (2014). Effect of thermal annealing on mechanical properties of polyelectrolyte complex nanofiber membranes. Fibers Polym..

[108] Liang Y., Cheng S., Zhao J., Zhang C., Sun S., Zhou N., Qiu Y., Zhang X. (2013). Heat treatment of electrospun Polyvinylidene fluoride fibrous membrane separators for rechargeable lithium-ion batteries. J. Power Sources.

[109] Ramaswamy S., Clarke L.I., Gorga R.E. (2011). Morphological, mechanical, and electrical properties as a function of thermal bonding in electrospun nanocomposites. Polymer.

[110] Lee S.J., Oh S.H., Liu J., Soker S., Atala A., Yoo J.J. (2008). The use of thermal treatments to enhance the mechanical properties of electrospun poly (ɛ-caprolactone) scaffolds. Biomaterials.

[111] Sheng J., Li Y., Wang X., Si Y., Yu J., Ding B. (2016). Thermal inter-fiber adhesion of the polyacrylonitrile/fluorinated polyurethane nanofibrous membranes with enhanced waterproof-breathable performance. Sep. Purif. Technol..

[112] Kancheva M., Toncheva A., Manolova N., Rashkov I. (2015). Enhancing the mechanical properties of electrospun polyester mats by heat treatment. Express Polym. Lett..

[113] Kaur S., Barhate R., Sundarrajan S., Matsuura T., Ramakrishna S. (2011). Hot pressing of electrospun membrane composite and its influence on separation performance on thin film composite nanofiltration membrane. Desalination.

[114] Gong W., Gu J., Ruan S., Shen C. (2019). A high-strength electrospun PPESK fibrous membrane for lithium-ion battery separator. Polym. Bull..

[115] Na H., Li Q., Sun H., Zhao C., Yuan X. (2009). Anisotropic mechanical properties of hot-pressed PVDF membranes with higher fiber alignments via electrospinning. Polym. Eng. Sci..

[116] Na H., Zhao Y., Zhao C., Zhao C., Yuan X. (2008). Effect of hot-press on electrospun poly (vinylidene fluoride) membranes. Polym. Eng. Sci..

[117] Lee S.W., Choi S.W., Jo S.M., Chin B.D., Kim D.Y., Lee K.Y. (2006). Electrochemical properties and cycle performance of electrospun poly (vinylidene fluoride)-based fibrous membrane electrolytes for Li-ion polymer battery. J. Power Sources.

[118] Lalia B.S., Guillen-Burrieza E., Arafat H.A., Hashaikeh R. (2013). Fabrication and characterization of polyvinylidenefluoride-co-hexafluoropropylene (PVDF-HFP) electrospun membranes for direct contact membrane distillation. J. Membr. Sci..

[119] He B., Tian L., Li J., Pan Z. (2013). Effect of hot-stretching on morphology and mechanical properties of electrospun PMIA nanofibers. Fibers Polym..

[120] Zhang L., Liu L.-g., Pan F.-L., Wang D.-F., Pan Z.-J. (2012). Effects of heat treatment on the morphology and performance of PSU electrospun nanofibrous membrane. J. Eng. Fibers Fabr..

[121] Wang X., Zhang K., Zhu M., Hsiao B.S., Chu B. (2008). Enhanced mechanical performance of self-bundled electrospun fiber yarns via post-treatments. Macromol. Rapid Commun..

[122] Elkhaldi R.M., Guclu S., Koyuncu I. (2016). Enhancement of mechanical and physical properties of electrospun PAN nanofiber membranes using PVDF particles. Desalin. Water Treat..

[123] Song Z., Hou X., Zhang L., Wu S. (2011). Enhancing crystallinity and orientation by hot-stretching to improve the mechanical properties of electrospun partially aligned polyacrylonitrile (PAN) nanocomposites. Materials.

[124] Hou X., Yang X., Zhang L., Waclawik E., Wu S. (2010). Stretching-induced crystallinity and orientation to improve the mechanical properties of electrospun PAN nanocomposites. Mater. Des..

[125] Wu S., Zhang F., Yu Y., Li P., Yang X., Lu J., Ryu S. (2008). Preparation of PAN-based carbon nanofibers by hot-stretching. Compos. Interfaces.

[126] Lai C., Zhong G., Yue Z., Chen G., Zhang L., Vakili A., Wang Y., Zhu L., Liu J., Fong H. (2011). Investigation of post-spinning stretching process on morphological, structural, and mechanical properties of electrospun polyacrylonitrile copolymer nanofibers. Polymer.

[127] Zhou Z., Lai C., Zhang L., Qian Y., Hou H., Reneker D.H., Fong H. (2009). Development of carbon nanofibers from aligned electrospun polyacrylonitrile nanofiber bundles and characterization of their microstructural, electrical, and mechanical properties. Polymer.

[128] Fennessey S.F., Pedicini A., Farris R.J. (2004). Mechanical Behavior of Nonwoven Electrospun Fabrics and Yarns.

